# KSHV episome tethering sites on host chromosomes and regulation of latency-lytic switch by CHD4

**DOI:** 10.1016/j.celrep.2022.110788

**Published:** 2022-05-10

**Authors:** Ashish Kumar, Yuanzhi Lyu, Yuichi Yanagihashi, Chanikarn Chantarasrivong, Vladimir Majerciak, Michelle Salemi, Kang-Hsin Wang, Tomoki Inagaki, Frank Chuang, Ryan R. Davis, Clifford G. Tepper, Kazushi Nakano, Chie Izumiya, Michiko Shimoda, Ken-ichi Nakajima, Alexander Merleev, Zhi-Ming Zheng, Mel Campbell, Yoshihiro Izumiya

**Affiliations:** 1 Department of Dermatology School of Medicine, University of California Davis (UC Davis), Sacramento, CA 95817, USA; 2 Lifescience Division, Lifematics, Osaka, Osaka 541-0046, Japan; 3 Tumor Virus RNA Biology Section, HIV Dynamics and Replication Program, National Cancer Institute, NIH, Frederick, MD 21702, USA; 4 Genome Center, Proteomics Core, Genome and Biomedical Sciences Facility, UC Davis, Davis, CA 95616, USA; 5 Department of Biochemistry and Molecular Medicine, School of Medicine, UC Davis, Sacramento, CA 95817, USA; 6 Department of Pathology and Laboratory Medicine, School of Medicine, UC Davis, Sacramento, CA 95817, USA; 7 Viral Oncology and Pathogen-Associated Malignancies Initiative, UC Davis Comprehensive Cancer Center, Sacramento, CA 95817, USA; 8 These authors contributed equally; 9 Lead contact

## Abstract

Kaposi sarcoma-associated herpesvirus (KSHV) establishes a latent infection in the cell nucleus, but where KSHV episomal genomes are tethered and the mechanisms underlying KSHV lytic reactivation are unclear. Here, we study the nuclear microenvironment of KSHV episomes and show that the KSHV latency-lytic replication switch is regulated via viral long non-coding (lnc)RNA-CHD4 (chromodomain helicase DNA binding protein 4) interaction. KSHV episomes localize with CHD4 and ADNP proteins, components of the cellular ChAHP complex. The CHD4 and ADNP proteins occupy the 5′-region of the highly inducible lncRNAs and terminal repeats of the KSHV genome together with latency-associated nuclear antigen (LANA). Viral lncRNA binding competes with CHD4 DNA binding, and KSHV reactivation sequesters CHD4 from the KSHV genome, which is also accompanied by detachment of KSHV episomes from host chromosome docking sites. We propose a model in which robust KSHV lncRNA expression determines the latency-lytic decision by regulating LANA/CHD4 binding to KSHV episomes.

## In brief

Kumar et al. perform Capture Hi-C to identify KSHV tethering sites on host chromosomes. They highlight that LANA interacts with CHD4, and KSHV episomes localize at CHD4 enriched regions. KSHV non-coding RNA binds and sequesters CHD4 during reactivation. This study furthers our understanding of the mechanism of the latency-lytic switch.

## Graphical abstract

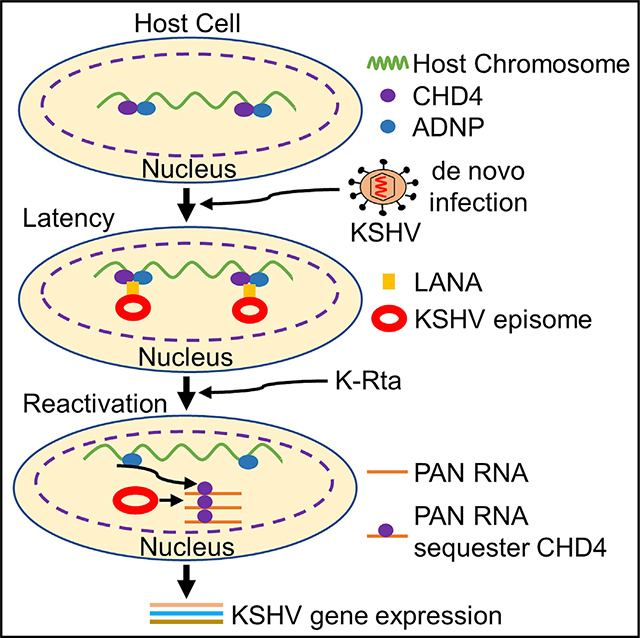

## INTRODUCTION

Kaposi sarcoma-associated herpesvirus (KSHV), discovered in 1994, is one of nine human herpesviruses (Chang et al., 1994). Since then, KSHV has been identified as the causative agent of Kaposi sarcoma (Chang et al., 1994; Ganem, 2010; Mesri et al., 2010) and two human lymphoproliferative diseases, primary effusion lymphoma (PEL) and AIDS-related multicentric Castleman disease (Cesarman and Knowles, 1999; Cesarman et al., 1995; Schulz, 2006; Wong and Damania, 2005). In cancer cells, KSHV establishes a latent infection in which most viral genes are silenced with the exception of several latent proteins, such as latency-associated nuclear antigen (LANA) (Kedes et al., 1997). KSHV establishes latency by continuous suppression of viral lytic gene expression (Yan et al., 2019). Various environmental factors, immunosuppression, unbalanced inflammatory cytokines, and other microbial co-infections have been shown to trigger KSHV reactivation (Aneja and Yuan, 2017).

KSHV LANA is a 130-kDa multifunctional protein that plays a major role in both DNA replication and episome maintenance during latency. LANA binds DNA sequences within the 801-base-pair terminal repeats (TRs) of the KSHV genome and plays a role for tethering episomes to the host cell chromosomes (Ballestas and Kaye, 2011; Hu et al., 2002; Stedman et al., 2004; Verma et al., 2006). There are two distinct mechanisms through which LANA tethers episomes to host cell chromosomes. First, the N terminus of LANA, which is predicted to be highly unstructured, can interact with host chromosomes through direct binding with histone H2A and H2B (Barbera et al., 2006; Piolot et al., 2001). A second mechanism involves the interaction of the LANA DNA binding domain (DBD) with host chromatin-associated proteins (Kim et al., 2013; Matsumura et al., 2010; Ottinger et al., 2006). The chromatin-associated proteins include BRD2 and BRD4, which contain two bromodomains that recognize the acetylated H3 and H4 histones, and a conserved C-terminal extraterminal domain (Wang et al., 2021; You et al., 2006). In both mechanisms, tethering requires a sequence-specific interaction between the LANA DBD and the viral episome at LANA binding sites 1 and 2 (LBS 1/2) (Hu et al., 2002). Episome maintenance requires at least two copies of LBS1/2 and the KSHV genome encodes 30 to 40 TRs, taking up to approximately one-fifth of KSHV genome (i.e., 24–32 kbp DNA fragment) (Lagunoff and Ganem, 1997). The crystal structures of the LANA DBD further revealed that LANA can form a higher-order decameric ring structure, and a hydrophobic interface between LANA dimers to form the decameric ring is important for cooperative LBS1/2 binding, DNA replication, and therefore episome maintenance (Domsic et al., 2013).

Chromatin remodeling enzymes have been of wide interest in the regulation of the KSHV latency-lytic switch. The enzymes include polycomb repressor complex 1 and 2, nucleosome remodeling and deacetylase (NuRD), and lysine demethylases (KDM2B, 3A, and 4A) (Gunther et al., 2019; Gunther and Grundhoff, 2010; Kim et al., 2013; Naik et al., 2020; Rossetto and Pari, 2012; Strahan et al., 2017; Toth et al., 2010). Recent studies highlight that chromatin remodeling enzymes also participate in regulation of genome architecture. In murine embryonic stem cells, the Iswi family remodeler Snf2h promotes CTCF binding to the genome and regulates formation of contact domains (Barisic et al., 2019; Clarkson et al., 2019). In addition to Snf2h, CHD4 (chromodomain helicase DNA binding protein 4) occupies and restricts enhancer accessibility as well as cohesin occupancy at CHD4 binding sites (Goodman et al., 2020). Genetic disruption of CHD4 therefore causes spontaneous differentiation concomitant with premature activation of lineage-specific genes (Arends et al., 2019; Ostapcuk et al., 2018; Sun et al., 2020). CHD4 has been identified in two distinct cellular protein complexes, the NuRD and ChAHP complexes (Farnung et al., 2020). The ChAHP complex consists of CHD4, ADNP (activity-dependent neuroprotective protein), and HP1γ (heterochromatin protein 1) (Ostapcuk et al., 2018). ChAHP complex was shown to modulate spatial chromatin loop organization locally (Kaaij et al., 2019), and plays essential roles in maintaining accurate cell fate decisions during development by regulating enhancer accessibility (Arends et al., 2019; Ostapcuk et al., 2018; Zhao et al., 2017). Accumulating evidence suggested that long non-coding RNAs (lncRNAs) also play a role in positioning of chromatin binding enzymes, linking local transcriptions with chromatin binding factor recruitments (Lu et al., 2021).

The KSHV genome encodes highly inducible lncRNAs, and those lncRNA promoters are direct targets of K-Rta (Ellison et al., 2009). The Poly Adenylated Nuclear RNA (PAN RNA) has produced significantly higher copies (up to 10^5^ copies) in a reactivating cell (Song et al., 2001; Sun et al., 1996). A recent report shows that PAN RNA could be replaced by other viral lncRNA sequences without a significant loss of KSHV replication, suggesting that there is a sequence-independent function (Withers et al., 2018).

In this study, we revealed KSHV episome tethering sites on host chromosomes with Capture Hi-C and characterized the surrounding nuclear microenvironment of KSHV episomes. The study suggested that expression of KSHV lytic genes is suppressed by CHD4. KSHV episomes colocalize with the CHD4 and ADNP on both host and KSHV chromosomes, and a CHD4 interaction with viral lncRNAs modulates binding of CHD4 on the KSHV genome. We propose a KSHV latency-lytic switch model, in which KSHV lytic inducible gene promoters are regulated by a balance between robust viral lncRNA expression and CHD4 complex tethering.

## RESULTS

### Identification of KSHV episome tethering site on host chromosomes

**Figure 1.**
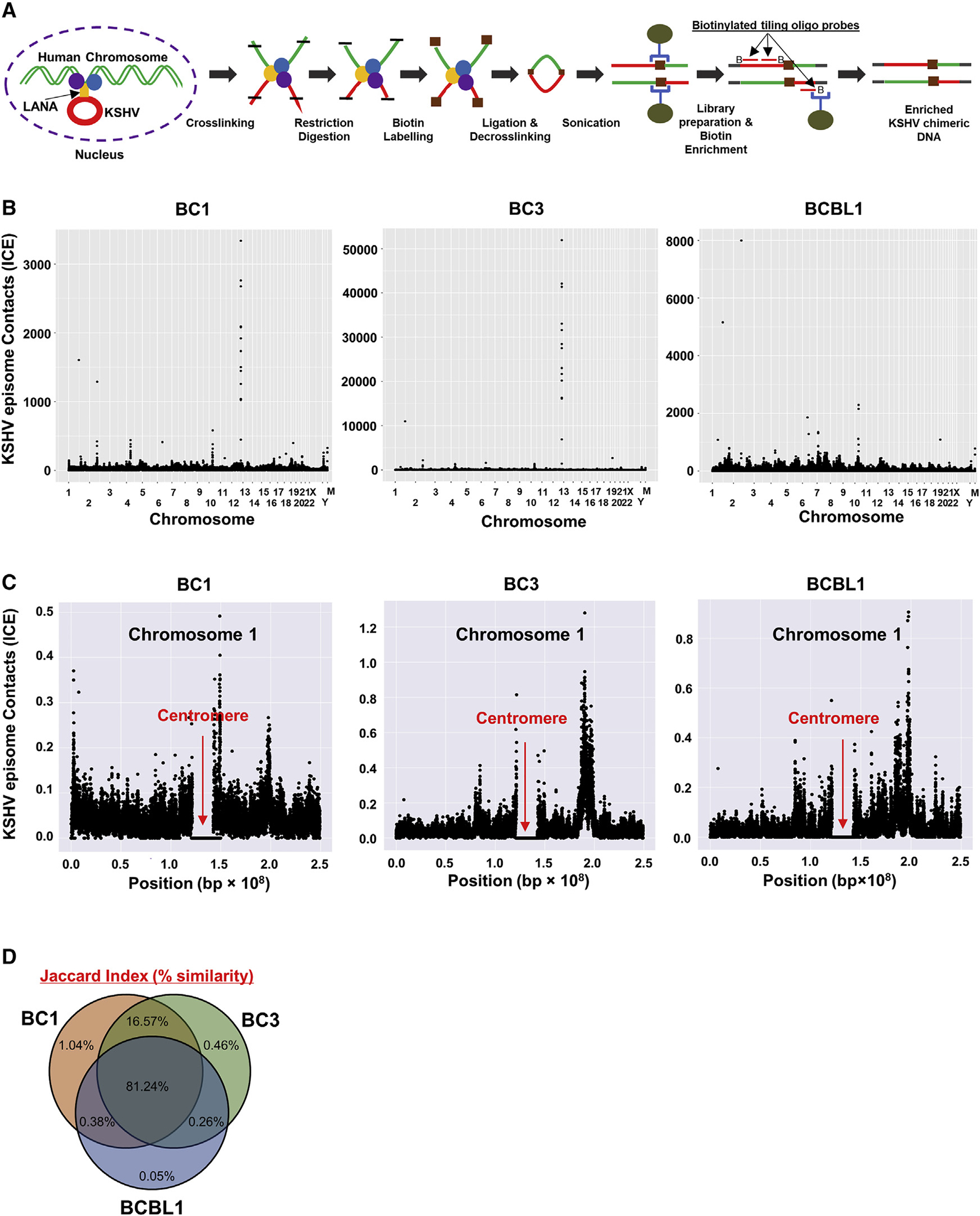
KSHV episome tethering sites in KSHV positive cell lines (A) Schematic workflow for Capture-HiC (CHi-C). (B) CHi-C chimeric DNA ligation products composed of sequences derived from the KSHV and human genomes were mapped in three naturally infected PEL cell lines. M, mitochondrial chromosome. ICE-corrected profiles depicting sums of filtered read counts binned at 10-kb resolution are shown. (C) Chromosome 1 dot plots are depicted and illustrate enrichment of sequence reads near the centromere. Red arrows indicate the position of the centromere. Extended panels for all other individual chromosome for three cell lines are presented in Figures S1A–S1C. (D) Venn diagram shows percent similarity of KSHV episome tethering positions among BC-1, BC-3, and BCBL-1 cells. (B–D) One biological replicate for BC-1 and BC-3, and three biological replicates for BCBL-1 with nearly identical results (similarity 0.95) (one representative is shown).

KSHV episomes tether to the host cell chromosomes via LANA; however, the mechanism of selection of docking sites is not well characterized. To understand how and where KSHV episomes tether to host chromosomes, we used the Capture Hi-C (CHi-C) to identify episome docking sites in three KSHV naturally infected PEL cell lines: BC-1, BC-3, and BCBL-1. The schematic diagram for the CHi-C procedure is presented in Figure 1A. We examined the position of host genomic regions that exhibited higher frequencies of normalized chimeric ligation products with KSHV DNA fragments and identified selectively enriched chimeric ligation read pairs throughout the host chromosomes (Figure 1B). The mapped reads on 23 individual chromosomes for BC-1, BC-3, and BCBL-1 are presented separately in Figures S1A–S1C. Each dot represents the number of normalized contact heterotypic sequence reads between host and KSHV sequences. The CHi-C normalized chimeric read counts on chromosome 1 for three naturally infected PEL cells are shown in Figure 1C. The results indicated that there are a higher number of chimeric read counts near centromeric regions in all three cell lines (Figure 1C). To study the frequencies of chimeric read counts in BC-1, BC-3, and BCBL-1 that are located near the centromere region, we performed a mathematic characterization, in which we extracted the chimeric read counts that derived from regions spanning a distance corresponding to 1% of the size of each chromosome at either 5′ or 3′ to centromeric chromosomal regions (marked green and blue) (Figure S1D). Counts per 100 kbp were calculated separately for each chromosome and compared to the average number across the individual chromosome. The results showed that higher chimeric read counts were observed at either 5′ or 3′ regions of the centromere. We also noticed that several chromosomes showed low numbers of chimeric read counts on both sides of the centromere (e.g., Chr9, Chr13), yet tethering patterns were similar among BC-1, BC-3, and BCBL-1 (Figure S1D). To further confirm the CHi-C findings, we performed DNA-fluorescence *in situ* hybridization (FISH) with centromere-specific peptide nucleic acid (PNA) probes in combination with LANA immunostaining. These results further showed that a large fraction of LANA dots were localized near centromeres (Figures S1E–S1G). The results are also consistent with a previous report, which demonstrated that LANA colocalized with the centromeric protein F (CENPF) and kinetochore protein, Bub1, and observed at centromere regions during metaphase (Xiao et al., 2010).

We next used a statistical measurement to examine whether KSHV docking sites are randomly distributed. For this, we examined the similarity of KSHV episome tethering sites using a Jaccard Index. We calculated the similarity of tethering sites based on the positions of chimeric sequence reads. The index identified 97.86% (BC-1 versus BC-3), 81.99% (BC-1 versus BCBL-1), and 82.36% (BC-3 versus BCBL-1) similarity (Figure 1D). These results demonstrated that a majority of KSHV episomes tether to similar host genomic regions in three naturally infected PEL cell lines, suggesting that there is a preferential nuclear microenvironment that can attract and maintain KSHV latent episomes.

### Identification of proteins in close proximity to LANA

**Figure 2.**
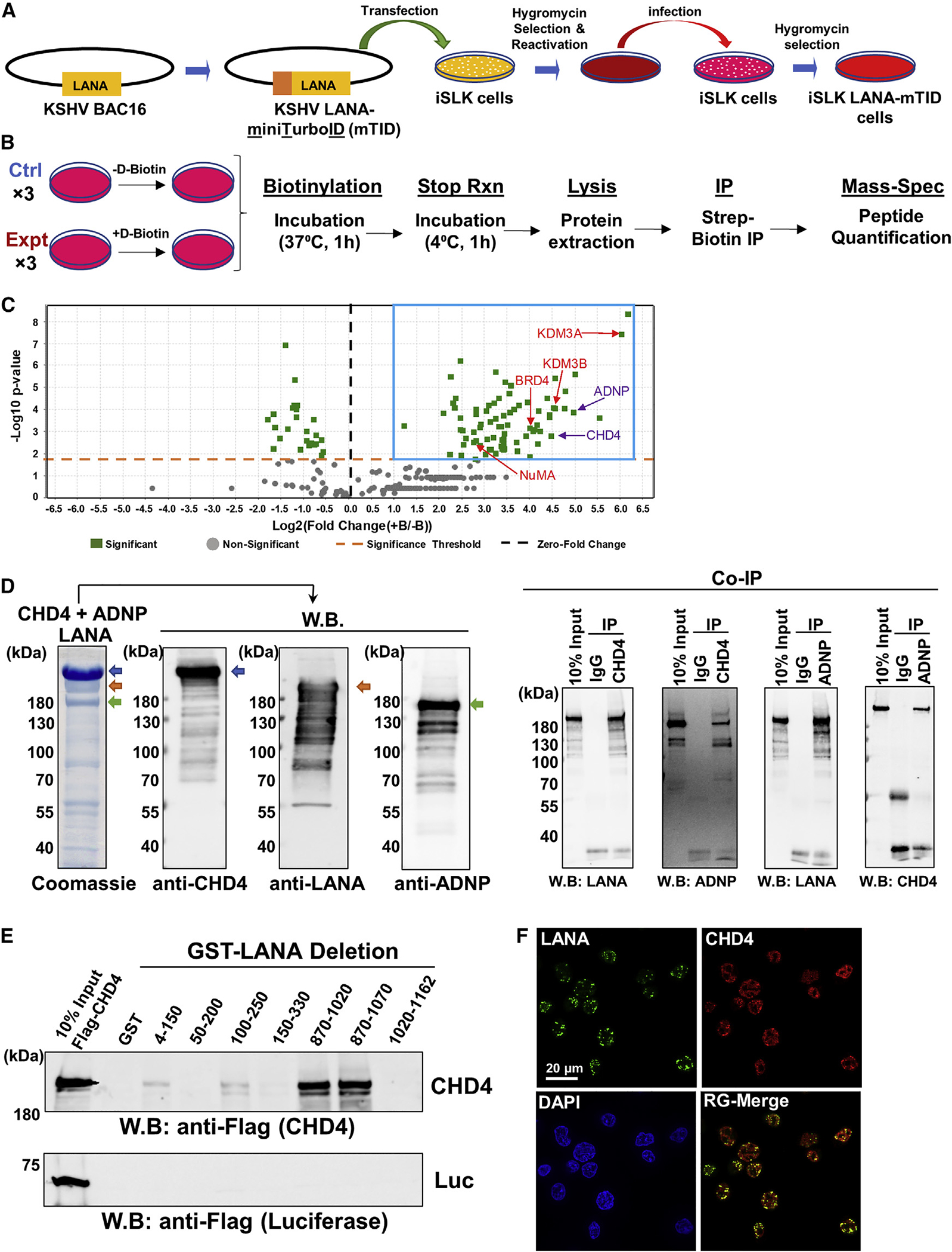
LANA interacts with the CHD4 and ADNP (A) A schematic diagram of preparation of recombinant KSHV-infected *i*SLK cells. (B) Experimental design for preparing samples for protein ID. *i*SLK-LANA mTID cells were left unincubated (−) or incubated (+) with D-biotin (500 μM) for 1 h. Unincubated (−) cells were used as control samples. (C) The volcano plot represents proteins identified in close proximity to LANA. Proteins with an abundance Log2 FC of greater than or equal to 1 and p value less than 0.05 were selected and are shown by blue box. Log2 FC was calculated as (+B/−B) where + B and −B indicate presence and absence of D-biotin, respectively. The t test was used for calculating the p value. Purple color, ChAHP components; Red color, previously known LANA interacting proteins. (D) LANA, CHD4, and ADNP complex, which consists of Flag-LANA (gold arrow), Flag-CHD4 (blue arrow), and His-ADNP (green arrow), was prepared by co-infected three recombinant baculoviruses, and the protein complex was isolated with FLAG-affinity purification. The authenticities of the respective protein bands were confirmed by immunoblotting with specific antibodies. Coomassie staining is shown. Ten percent of the reaction before immunoprecipitation was used as controls. W.B., western blotting; CoIP, Co-immunoprecipitation. (E) An equal amount (1 μg) of each LANA deletion protein purified from *E. coli* was incubated with full-length Flag-tagged luciferase (1 μg) or Flag-tagged CHD4 (1 μg) in binding buffer and interaction was probed with anti-Flag antibody. LANA deletion proteins are presented in Figure S2C. (F) CHD4 and LANA were probed with anti-CHD4 rabbit monoclonal antibody and anti-LANA rat monoclonal antibody, respectively. Images were acquired with Keyence fluorescence microscopy. Scale bar, 20 μm. (D–F) n = 3 biological replicates, and one representative is shown. (C) Each protein ID was performed with three biological replicates.

Next, we examined the nuclear protein microenvironment of KSHV episome tethering sites in infected cells. We hypothesized that, by examining cellular proteins neighboring to LANA in infected cells, we may identify the repertoire of proteins important for selection of KSHV episome docking sites. To identify proteins in close proximity to LANA, we employed a proximity biotin labeling approach (Kumar et al., 2021). The mini-TurboID is a biotin ligase that covalently attaches biotin to lysine residues in neighboring proteins (<10 nm) (Branon et al., 2018; Kumar et al., 2021). A recombinant KSHV BAC16 with LANA N-terminally tagged with mini-TurboID (referred to as KSHV LANA-mTID) was prepared; the procedure for the preparation of KSHV LANA-mTID is presented in Figure S2A. We transfected *i*SLK cells with the recombinant KSHV LANA-mTID and stably selected with hygromycin (1 mg/mL). The KSHV LANA-mTID virus was then recovered by inducing reactivation with doxycycline and sodium butyrate for 5 days. Similarly, we also recovered wild type (WT) KSHV virus from WT-BAC16 KSHV containing *i*SLK cells and compared the infectivity of LANA-mTID KSHV with WT-BAC16 KSHV. The results showed that PAN RNA and LANA expression in mTID-LANA infected cells was slightly less than the WT-BAC16 KSHV. These results suggest that mTID-LANA virus is still infectious; however, the mTID tag may interfere with virus lytic gene expression to a certain degree during *de novo* infection (Figure S2B). With LANA-mTID KSHV infectious particles, the *i*SLK cell line was infected and selected with hygromycin to generate stable *i*SLK-LANA mTID cells (Figure 2A). The *i*SLK-LANA mTID cells were incubated with D-biotin for 60 min in culture media (Figure 2B). This strategy identified 76 host proteins (p < 0.05) that were physically neighboring KSHV LANA within a 10-nm radial distance during the period of D-biotin incubation (Branon et al., 2018). These proteins included nuclear mitotic apparatus protein (NuMA), bromodomain-containing protein 4 (BRD4), and lysinespecific demethylase 3A and 3B (KDM3A and 3B) that have been previously shown to interact with LANA (Hellert et al., 2013; Kim et al., 2013; Si et al., 2008; Vermaetal., 2013) (Figure2C; Table S1). The study also precipitated components of the ChAHP complex (Ostapcuk et al., 2018), which is composed of chromodomain helicase DNA binding protein 4 (CHD4), ADNP, and HP-1γ with high confidence (Figure 2C). Although we could not identify HP-1γ with our statistical criterion in proteomics study, HP-1γ protein was previously shown to interact with KSHV LANA (Lim et al., 2003). On the other hand, we also found several proteins that exhibited decreased interaction in the presence of biotin; we speculate that those proteins were binding to the streptavidin beads non-specifically. The LANA interaction with CHD4 and ADNP was further validated with *in vitro* pulldown assays after purifying individual components from recombinant baculovirus-infected cells (Figure 2D). Further, recombinant GST-tagged LANA deletion proteins were used to map the interaction domain with CHD4, and found that the amino acid (aa) residues 870 to 1,070, near the LANA DBD, were responsible for interaction with CHD4 (Figures 2E and S2C). Immunofluorescence assays with mono-specific antibodies further confirmed that LANA and CHD4 were colocalized in naturally infected BCBL-1 cells (Figure 2F). Taken together, these results suggest that LANA associates with CHD4 and ANDP in latently infected cells.

### Association of KSHV episome tethering sites with CHD4 and ANDP binding

**Figure 3.**
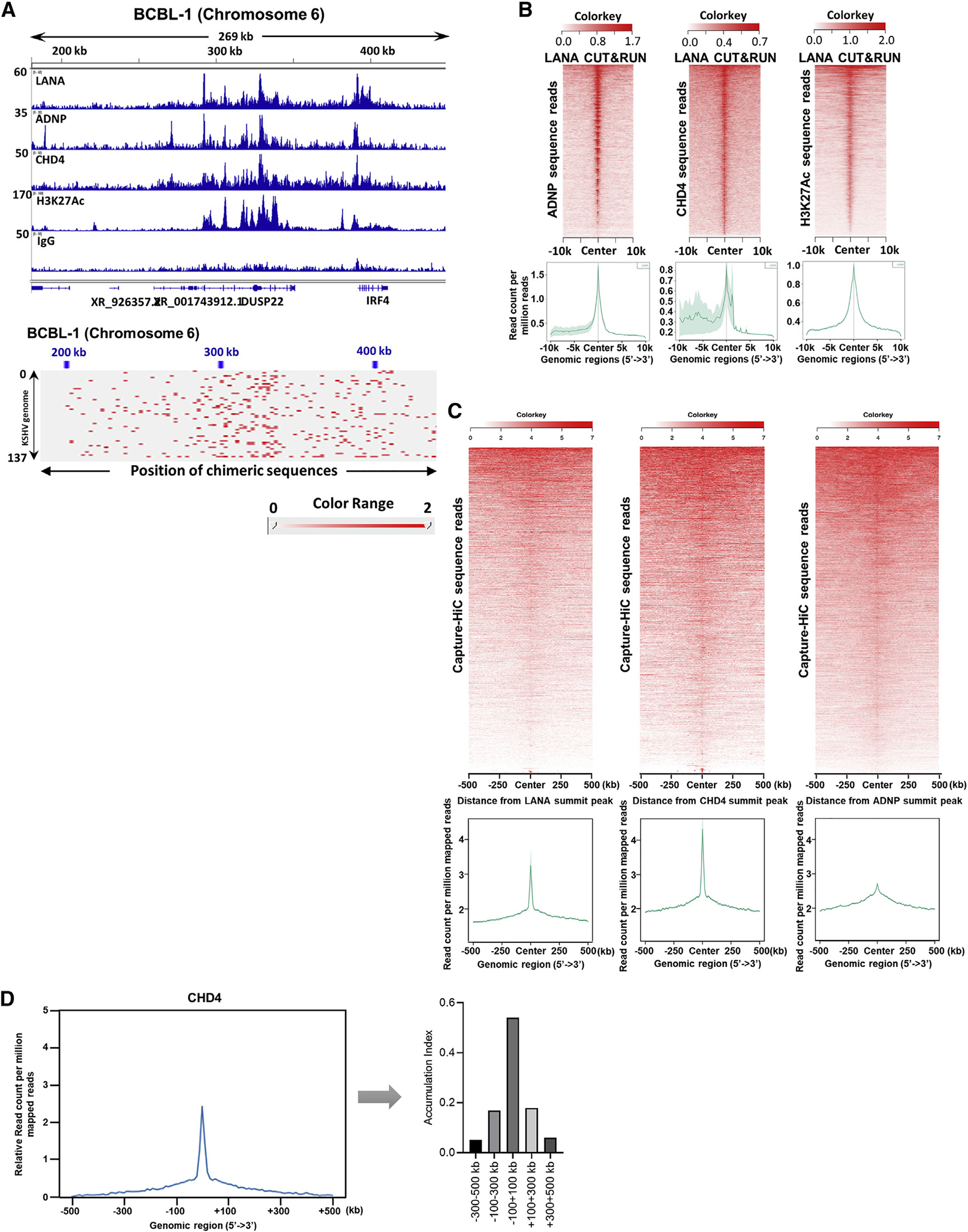
Association of LANA-ADNP-CHD4 complex binding with KSHV episome tethering sites in the human genome (A) CUT&RUN was performed for the indicated proteins. Peaks were visualized with Integrative Genomics Viewer (IGV) and one of the major binding sites (IRF4 enhancer region) is shown. The height of the peak (e.g., read depth) and positions on chromosome 6 are denoted on the left-hand side and top of the panels, respectively. The bottom panel shows the mapped chimeric sequence reads between KSHV and host genomic loci visualized with Juicebox. (B) Heatmap (top) and average profile (bottom) showing correlation of LANA enrichment (by color intensity and region) with ADNP, CHD4, and H3K27Ac occupancies. Average profile plot summarizing the heatmap (bottom). The lighter green shade represents the standard error (SEM) on the average profile plot. Color keys are shown on the each heatmap (top). The x axis shows the distance from LANA summit peaks. (C) The association between CHi-C chimeric reads pair products and LANA, CHD4, and ADNP CUT&RUN peaks were depicted as heatmap and average profile plot. (D) The accumulation of CHD4 is displayed as a proportion of area under the curve of the relative average profile. Association between distance from CHD4 summit peak and relative chimeric sequence counts is shown as a bar chart. (A) n = 2 biological replicates for each CUT&RUN, one representative is shown.

The protein interaction and colocalization in the nucleus led us to further investigate the localization of CHD4, ADNP, and LANA on both host and KSHV chromosomes. To identify the chromatin occupancy site(s) for CHD4, ADNP, and LANA, we employed Cleavage Under Targets and Release Using Nuclease (CU-T&RUN) (Skene and Henikoff, 2017). The LANA, CHD4, and ADNP CUT&RUN peaks clearly overlapped at multiple sites of cell host chromosomes primarily with active histone marks (H3K27Ac), which include previously described IRF4 enhancer regions (Manzano et al., 2020; Wang et al., 2020) (Figure 3A, top). In addition, higher frequencies of chimeric products with KSHV genomic DNA sequences were also identified in the genomic region (Figure 3A, bottom). To further examine the degree of colocalization of CHD4 and ADNP at regions containing LANA binding sites, all CHD4 or ADNP coverage around LANA CUT&RUN summit peaks were ranked by their enrichment levels and aligned in descending order within a 10-kb window. Heatmaps (Figure 3B, top) and average enrichment profiles (Figure 3B, bottom) were generated with NGS plot (Shen et al., 2014) and the results demonstrated that the co-occupancies of LANA, CHD4, and ADNP. Because these proteins were indicated to interact with each other in *in vitro* binding studies (Figures 2D and 2E), we hypothesized that the LANA/CHD4/ADNP complex may also play a role in tethering KSHV episomes to host chromosomes. To test this, CHi-C chimeric reads were aligned around LANA, CHD4, and ADNP CUT&RUN summit peaks, respectively, and visualized in descending order at a 500-kb window. The results showed that KSHV chimeric sequence reads with the host genome (y axis) were primarily localized in proximity to LANA, CHD4, and ADNP CUT&RUN signals (Figure 3C). To further calculate the degree of interaction, we measured the relative CHi-C chimeric reads per million and examined the association with relative distance to CHD4 binding sites. Accumulation index calculated from the NGS plot suggested that more than 50% of CHi-C reads are closely located at CHD4 binding within a 200-kb window (Figure 3D). Altogether, these results suggest that KSHV episomes tether to host chromosome at CHD4-and ADNP-enriched sites.

### KSHV LANA colocalization with CHD4 and ADNP on KSHV episomes

**Figure 4.**
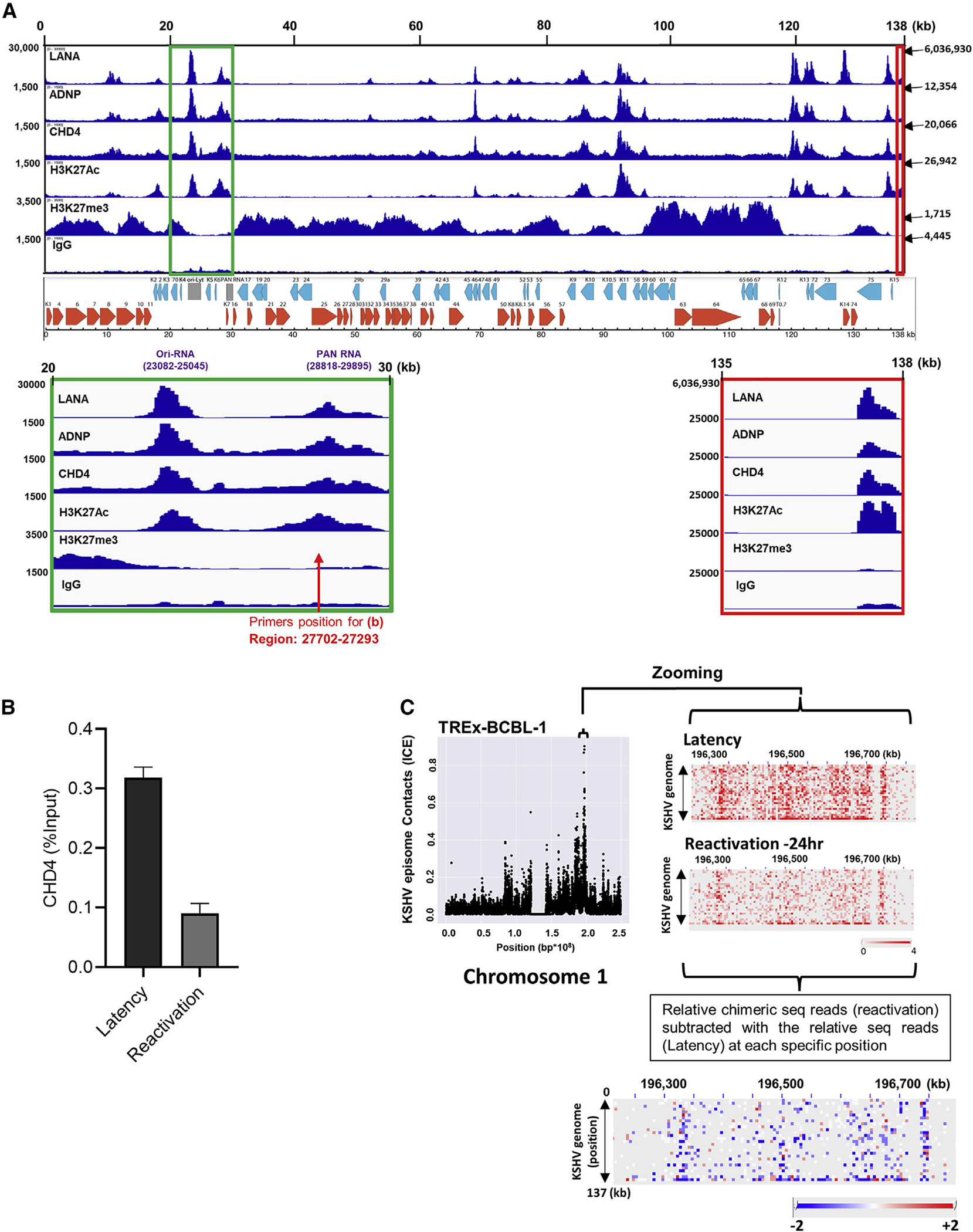
LANA, CHD4 and ADNP colocalize on the KSHV genome (A) The indicated antibodies were used for CUT&RUN in BCBL-1 cells and sequence reads were mapped to the KSHV genome. IGV snapshots and KSHV genome map are shown. The height of the peak (e.g., read depth; left-hand side of panel), position on KSHV genome in kilobase (kb; top of panel), and numbers indicated by arrow (right-hand side of panel) denote the height of the peak on terminal repeat region (TR). The green box (20- to 30-kb region) and red box (135- to 138-kb region) are zoomed in to show Ori-RNA, PAN RNA, and TR region, respectively. The positions of primers used in Figure 4B are also shown with red arrows. (B) TREx-BCBL-1 cells were reactivated with doxycycline (1 μg/mL) and TPA (20 nM) for 24 h. CUT&RUN was performed on un-reactivated (latency) and reactivated cells for CHD4. CHD4 binding on the PAN RNA promoter region (red arrow in Figure 4A) was calculated relative to the input sample. (C) TREx-BCBL-1 cells were reactivated with doxycycline (1 μg/mL) and TPA (20 nM) for 24 h. Chimeric sequence reads for un-reactivated (latency) and reactivated cells were mapped to the host chromosomes and visualized by Juicebox. Relative tethering for the selected region on chromosome 1 was calculated by subtracting relative chimeric sequence reads in latency from relative chimeric sequence reads during reactivation. Blue and red dots indicate decreased and increased chimeric sequence reads, respectively. The data shown are representative of (A) n = 2, (B) n = 3, and (C) n = 3, biological replicates. (B) qPCR with three technical replicates is shown and data are presented as mean ± SD.

We next examined CHD4 and ADNP binding sites on the KSHV episome. The results again showed colocalization among LANA, CHD4, and ADNP with the active histone mark, H3K27Ac and exceptionally strong peaks were seen at terminal repeat regions (Figure 4A), where multiple copies of LANA bind (Garber et al., 2001). The viral lncRNAs, especially PAN RNA, are known to be expressed at higher transcript copy numbers than the open reading frames during lytic replication (Purushothaman et al., 2015; Song et al., 2001), and CHD4, ADNP, and LANA were enriched at the 5′ regions of these lncRNA promoter regions (Figures 4A and S3A). Next, the effects of KSHV reactivation on CHD4 occupancies were examined by CUT&RUN with qPCR. The results suggested that CHD4 occupancies on the KSHV genome were reduced during KSHV reactivation (Figure 4B). We also examined effects of KSHV reactivation on the KSHV episome tethering on host cell chromosomes. For this, TREx-BCBL-1 cells were reactivated for 24 h and CHi-C samples were prepared. We measured the relative chimeric DNA sequence frequencies of KSHV with host chromosomes before and after KSHV reactivation. To reveal differences of relative sequence reads at one of the KSHV episome tethering sites, normalized sequence reads in latent samples were subtracted from those in reactivated samples. The results showed decreased KSHV chimeric sequence reads, suggesting that KSHV reactivation induced detachment of the KSHV episome from host chromosomes (Figure 4C). There was no measurable increase in KSHV genomic DNA, suggesting that observed effects were unlikely due to increased KSHV genomic copy number by KSHV DNA replication. Whereas, at same time point, PAN RNA expression was strongly induced (Figure S4A).

### Identification of PAN RNA binding proteins with proximity biotin ligation

**Figure 5.**
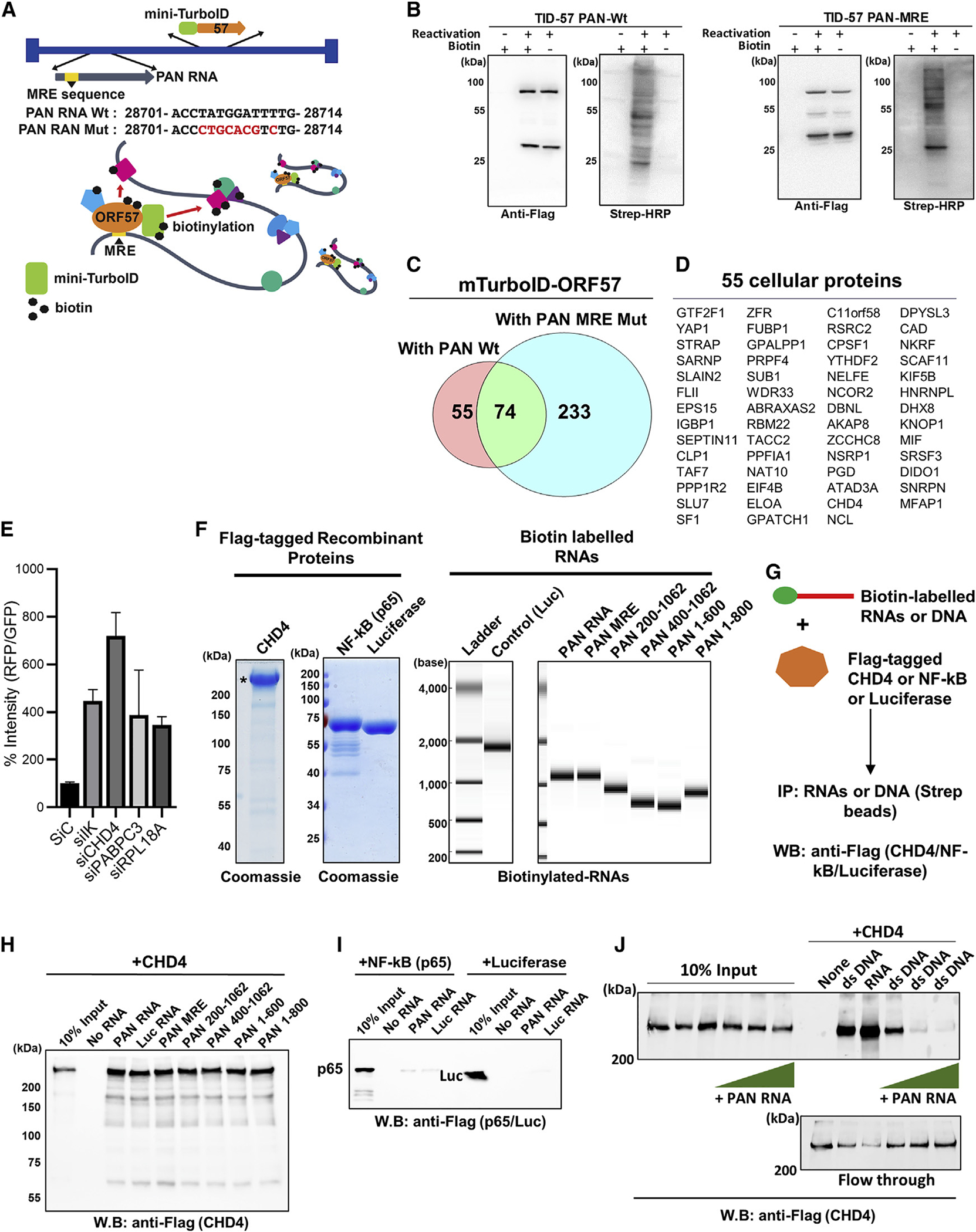
CHD4 is an RNA and DNA binding protein (A) A schematic diagram of recombinant KSHVs. (B) Recombinant KSHV-infected *i*SLK cells (TID-57 PAN Wt and MRE) were either untreated or induced for reactivation with doxycycline (1 μg/mL) and TPA (20 nM) for 24 h. Blots were probed with either anti-Flag antibody (for mini-TurboID-ORF57) or Streptavidin-HRP (for biotinylated proteins). (C) The Venn diagram indicates the number of identified proteins with a p value less than 0.05. (D) The proteins found enriched in PAN RNA WT samples are shown. The entire list of proteins and peptide counts are presented in Table S2. (E) Recombinant KSHV reactivation, which encodes RFP under control of the PAN RNA promoter, was used to screen the effects of KSHV reactivation. RFP signal intensity was measured with ImageJ, and the GFP signal was used as internal controls. Three randomly selected fields in the middle of each well were quantified and the average intensity was plotted. (F) Recombinant Flag-tagged proteins were expressed with baculovirus and purified with Flag-agarose beads. Coomassie staining of Flag-CHD4, Flag-NF-kB (p65), and Flag-luciferase used for pulldown studies are shown. (G) Schematic for *in vitro* interaction assay performed in (H–J). (H) RNA pulldown was performed with the indicated biotinylated PAN RNA deletions, mutation (MRE), and irrelevant RNA (luciferase mRNA), and interaction was probed by immunoblotting with anti-Flag antibody. Beads alone (No RNA) was used for background control. (I) RNA pulldown was performed with indicated biotinylated RNAs, and p65 and luciferase (Luc) were visualized by using anti-Flag antibody. (J) Pulldown analyses with biotinylated ssRNA or dsDNA was performed. CHD4 (100 nM) was incubated with biotinylated RNA (100 nM) or biotinylated dsDNA (100 nM) in 40-μL binding buffer. Increasing amounts of non-biotinylated PAN RNA at 1:1, 1:10, and 1:20 (dsDNA versus ssRNA) were also incubated, and precipitated CHD4 protein in the pulldown was probed with anti-Flag antibody. Flow-through and 10% of the input reaction before pulldown were used as control. (B, E, F, and H–J) n = 3 biological replicates, and one representative is shown. (E) Data are presented as mean ± SD. (C) Each protein ID was performed with three biological replicates.

Previous studies suggested that PAN RNA transcription *in cis* plays an essential role in initiation of KSHV lytic replication. Inhibition of PAN RNA transcription by either deletion of the PAN RNA sequence or mutation at K-Rta responsive elements in the PAN RNA promoter significantly impaired distantly located promoter activation and KSHV replication (Campbell et al., 2018; Rossetto et al., 2013). In addition, CHD4 is known to localize to cellular enhancers to regulate enhancer-promoter interactions (Arnold et al., 2019). To examine PAN RNA-mediated transcriptional regulation, we again applied the proximity labeling technique to profile PAN RNA neighboring proteins during KSHV reactivation. This time, the mTID cassette was first inserted as an N-terminal fusion of ORF57, a PAN RNA binding protein, and the fusion protein was expressed from the endogenous promoter during KSHV reactivation (Figure 5A). Taking advantage of a previous detailed mapping study, which identified ORF57 binding sites on PAN RNA, termed MRE (Mta Responsive Element) (Massimelli et al., 2011), we used a PAN MRE mutant virus having a 9-nucleotide mutation in the MRE element (Figure 5A). KSHV genome-wide qRT-PCR array analysis confirmed previous studies that the PAN RNA MRE mutation impaired viral gene expression, with stronger effects being seen in late gene cluster regions (Figure S5A right panel). We also confirmed that protein biotinylation occurred only in the presence of both ORF57 protein (reactivated sample) and biotin (Figure 5B). By comparing the enrichment profiling between the PAN RNA MRE mutant and PAN RNA WT, we isolated cellular proteins that are in proximity to ORF57 protein via PAN RNA binding. Proximity biotin labeling identified a total of 129 and 307 proteins from mTID-57 PAN WT *i*SLK cells and mTID-57 PAN MRE *i*SLK cells, respectively (p < 0.05) (Table S2). Among the interacting proteins, 74 proteins were in common between the PAN MRE WT and PAN MRE mutant, while 55 proteins were enriched in the presence of the WT PAN RNA sequence (Figures 5C and 5D). Deletion of MRE seemed to unleash ORF57 protein and allow ORF57 to interact more freely with other RNA binding proteins (Figure 5C). Importantly, this proteomics approach identified CHD4 as a putative PAN RNA binding protein (p < 0.005). A small interfering RNA (siRNA) screening of 129 (55 + 74) PAN RNA binding proteins was performed to examine effects on KSHV reactivation with a reporter cell line, *i*SLK.219. The screening identified four strong repressors: IK, CHD4, PABPC3, and RPL18A with fold change (RFP/GFP) >3 among the 129 proteins, and CHD4 was found to be the strongest among them (RFP/GFP >7-fold) (Figure 5E). Gene Ontology analysis of 129 PAN RNA-mediated ORF57-interacting proteins suggested that ORF57 is indeed primarily involved in RNA processing (Figure S5B).

### A viral lncRNA inhibits CHD4 double-stranded DNA binding

PAN RNA MRE-dependent enrichment by ORF57-mediated biotinylation suggested that CHD4 is an RNA binding protein, and that PAN RNA brings the CHD4 and ORF57 proteins into proximity by serving as a scaffold for biotinylation (model shown in Figure 5A). In order to examine the interaction of CHD4 with PAN RNA, we prepared purified RNAs (PAN RNA, MRE mutant, PAN RNA deletion mutants, and luciferase RNA) and proteins (CHD4, nuclear factor (NF)-kB, and luciferase) (Figure 5F), and performed *in vitro* interaction assays (depicted in Figure 5G). The results showed that CHD4 was indeed precipitated with PAN RNA; however, MRE mutant, PAN RNA deletion mutants, as well as irrelevant luciferase RNA also interacted with CHD4, suggesting that CHD4 RNA binding is unlikely sequence-specific under our binding conditions (Figure 5H). However, the same RNA binding conditions with full-length PAN RNA, did not precipitate a DNA binding protein, NF-κB (p65) nor luciferase protein, suggesting that CHD4 does possess RNA binding capacity (Figure 5I). Because a previous study showed that *Drosophila melanogaster* CHD4 homolog is capable of binding DNA, and human CHD4 ATPase activity is stimulated in the presence of naked DNA (Morra et al., 2012), we tested if purified CHD4 protein is able to bind to a double-stranded DNA (dsDNA) fragment encoding the PAN RNA sequence. The results showed that CHD4 was able to bind dsDNA directly (Figure 5J). Importantly, increasing amounts of PAN RNA (single-stranded RNA [ssRNA]) antagonized CHD4 dsDNA binding, which was completely blocked in the presence of non-biotinylated PAN RNA at a 1:10 (dsDNA/RNA) molecular ratio (Figure 5J). The results suggest that locally transcribing PAN RNA (which would reach up to 3 × 10^5^ copies/cell [Song et al., 2001]) may function to sequester CHD4 away from the KSHV genome, which is seen by reduced CHD4 on the KSHV genome (Figure 4B).

### CHD4 is important for latency maintenance and establishment

**Figure 6.**
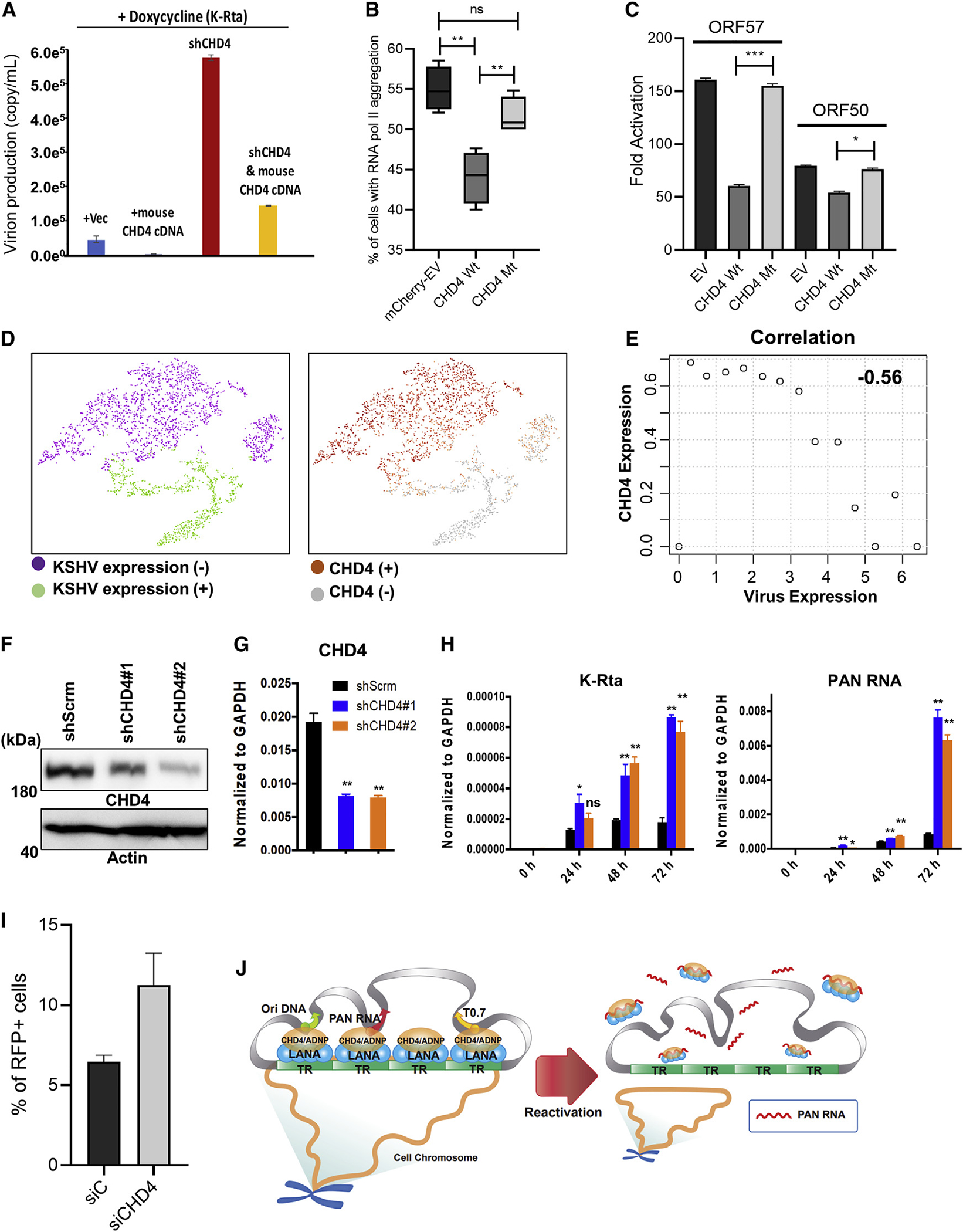
CHD4 is important for latency maintenance and establishment (A) *i*SLK.219 cell line was transfected with empty vector (Vec), mouse Chd4 cDNA, human CHD4 shRNA, or CHD4 shRNA plus mouse Chd4 cDNA for 48 h followed by KSHV reactivation with doxycycline (1 μg/mL) for 5 days. ORF57 plasmid was used to generate a standard curve and encapsidated KSHV genome copy number was measured by qPCR. (B) *i*SLK cells latently infected with BAC16-Wt was transfected with mCherry empty vector (EV) or Flag-tagged mouse wild type CHD4 (CHD4 Wt), or an ATPase domain mutant (CHD4 Mt). The percentage of RNA pol II aggregate formation in mCherry positive or Flag-positive cells was measured and plotted. Fifty cells were counted in each of the three biological replicates. Two-tailed unpaired t test was performed. **p < 0.01, ns, non-significant. (C) *i*SLK.219 cells were transduced with an equal amount of indicated lentivirus for 2 days. Cells were reactivated with doxycycline (1 μg/mL) for 2 days and ORF50 and ORF57 gene expression was quantified by RT-qPCR. Untreated cells were used for calculating the relative expression of ORF50 and ORF57. GAPDH was used as internal controls. Two-tailed unpaired t test was performed. *p < 0.05, ***p < 0.001 (comparing with CHD4 Wt). (D and E) *i*SLK.219 cells were reactivated by induction of K-Rta expression from a doxycycline inducible promoter for 24 h and single-cell sequencing was performed. (D) tSNE was applied to separate cell clusters and the expression of KSHV genes (D, left panel) and CHD4-expressing cells were marked (D, right panel). (E) Cells were divided into groups based on the amount of KSHV transcripts. A table and distribution of cell numbers and amount of KSHV transcripts are shown in Figures S6A and S6B. A correlation was established based on CHD4 expression in different groups based on KSHV transcript levels. (F–H) 293FT cells were transduced with scramble shRNA (shScrm) or shCHD4 and stably selected with antibiotic. Immunoblot (F) and RT-qPCR (G) analysis confirmed CHD4-KD. 293FT stable cells were infected with rKSHV.219 for 24 h and total RNA was harvested at the indicated time points. (H) K-Rta and PAN RNA was measured by RT-qPCR and normalized to GAPDH. *p < 0.05, **p < 0.01 (comparing with shScrm). (I) 293FT cells were transfected with control siRNA (siC) or CHD4 siRNA (siCHD4) for 48 h followed by infection with rKSHV.219 virus for 96 h. Flow cytometry analysis was performed to calculate the percentage of RFP-positive cells. (J) CHD4-mediated latency-lytic switch model. KSHV episomes tether at regions of CHD4/ADNP/LANA complex localization. KSHV reactivation triggers robust PAN RNA expression, PAN RNA molecule competes with CHD4 DNA binding, which enhances KSHV lytic gene transcriptions. Inhibition of CHD4 DNA binding may also contribute to detachment of KSHV episomes from CHD4-enriched host chromosome regions. (A–C, F–I) n = 3 and (D and E) n = 1 biological replicates, and one representative is shown. (A, C, G, H) qPCR with three technical replicates is shown. (A–C, G–I) Data are presented as mean ± SD.

The studies above indicate that LANA interacts with the CHD4 complex on both cellular and viral chromosomes, and that CHD4 may restrict KSHV PAN RNA expression and hence KSHV lytic reactivation. Accordingly, we next examined the significance of CHD4 in the KSHV latency-lytic switch. CHD4 expression in the *i*SLK.219 cell was knocked down with small hairpin RNA (shRNA) and the degree of KSHV reactivation was examined after triggering K-Rta expression with doxycycline. The results showed that knockdown of CHD4 enhanced KSHV replication more than 8-fold over K-Rta induction alone. Conversely, functional re-introduction of CHD4 by overexpression of mouse *Chd4* cDNA (i.e., in order to escape from the shRNA, which specifically targets human CHD4) counteracted effects of CHD4 knockdown (Figure 6A). Notably, overexpression of mouse Chd4 almost completely abolished K-Rta-mediated KSHV reactivation (Figure 6A, second bar), and inhibited the aggregation of RNAPII on the KSHV genome as measured by immunostaining with overexpressed CHD4 and RNAPII (Figure 6B). Strong silencing effects were dependent on CHD4’s ATPase activity since mutations in the helicase domain, using the same mutation found in patients with CHD4-associated syndrome (Farnung et al., 2020; Weiss et al., 2020), did not reduce RNAPII aggregation and KSHV transcription (Figures 6B and 6C). In addition to the knockdown or overexpression studies, we also performed single-cell transcriptomic studies with reactivated *i*SLK.219 cells and examined the association of cells with successfully triggered reactivation with the amount of cellular *CHD4* transcripts. For this, KSHV transcripts were extracted and sorted based on sequence counts and examined for correlation with cellular gene expression at the single-cell level (Figures S6A and S6B). The results indicated that the presence of higher levels of CHD4 transcripts showed inhibitory effects for either the triggering of reactivation and/or prolonging the lytic viral gene transcription burst (Figures 6D and 6E), while a similar negative correlation was not observed for *GAPDH* or the similarly expressed *HDAC2*, a component of the NuRD complex, another CHD4 complex (Figure S6C).

Finally, the significance of CHD4 in the establishment of latency was examined. We first knocked down CHD4 in 293T cells and infected them with purified KSHV r.219 virus to monitor viral gene silencing. CHD4 knockdown was confirmed at the protein and RNA levels (Figures 6F and 6G). The results showed that KSHV gene expression continued to increase during a 3-day period in two independent CHD4 KD cells, while KSHV gene expression did not increase in control shScramble cells (Figure 6H). Further, KSHV lytic replication was also monitored with RFP signals within the cell population, and the results demonstrated that the number of RFP-positive cells was higher in the CHD4 knockdown 293T cells (11.25%) compared with the negative control knockdown (siC) cells (6.45%) at 96 h post infection (Figures 6I, S6D and S6E). Altogether, these results suggest that CHD4 may function to silence KSHV lytic genes at an early stage of KSHV *de novo* infection and facilitate both the entry and maintenance of latency, perhaps by suppressing robust viral lncRNA expression, which locally accumulates active RNAPII complexes for inducible other lytic gene promoters.

## DISCUSSION

Recent exciting studies suggest that an RNA binding domain in transcription factor CTCF is essential for the formation of substructures of genome architecture (Hansen et al., 2019; Saldana-Meyer et al., 2019). The presence of distinct classes of RNA binding protein (RBP)-dependent genomic loop formation suggests the participation of nascent RNAs and other RBP partners in regulation at transcriptionally active genomic loci. It is also known that lncRNAs and RNA binding proteins participate in transcriptional factories (Arnold et al., 2019; Isoda et al., 2017; Nair et al., 2019). In herpes simplex virus 1 infected cells, the viral genomes remain free of cellular nucleosomes, which increases the accessibility for RNAPII and other DNA binding proteins to establish transcription factories (McSwiggen et al., 2019). Another study by Dr. DeLuca’s group has demonstrated that HSV-1 transcription activator, ICP4, is responsible for recruiting cellular RNAPII to the cellular nucleosome-free viral genome (Dremel and DeLuca, 2019). Here, we show that overexpression of CHD4 prevents RNAPII aggregate formation at chromatinized-KSHV latent episomes and inhibits KSHV reactivation. Consistent with CHD4 being a strong repressor, loss-of-function mutations induce enhancer activation leakage, which associates with multiple developmental disorders in humans (Coe et al., 2019; Kovac et al., 2018; Le Gallo et al., 2012; Sifrim et al., 2016; Zhao et al., 2013, 2017).

CHD4 is known to form two distinct protein complexes, ChAHP and NuRD. Although we did not identify components of the NuRD complex in our proximity biotin labeling, it is possible that a small fraction of LANA still interacts with NuRD complex and regulates KSHV reactivation (Naik et al., 2020). The siRNA screening for epigenetic factors, which have an impact on KSHV replication, has been reported (Naik et al., 2020). The report showed that CHD4 is indeed a suppressor for KSHV lytic replication. Here, we complemented the finding that KSHV episomes are localized with CHD4 and ADNP on both KSHV and host chromatins, and interact with LANA at the hydrophobic interface of LANA dimers near the LANA DBD (Correia et al., 2013; Domsic et al., 2013). Suppression of KSHV lytic genes by CHD4 in *de novo* infected cells also suggests that there might be active recruitment of the CHD4 to the incoming KSHV genomes. The mechanism(s) that enable KSHV episomes to select specific CHD4 binding sites on host chromosomes, and whether these CHD4-occupied host genomic regions are linked to tissue-specific gene expression, have yet to be characterized. At least in the three PEL cell lines examined, one of the common CHD4/ADNP/LANA complex-recruitment sites is IRF4 enhancer region, which is highly active in the B cell lineage (Carbone et al., 2000; Manzano et al., 2020; Wang et al., 2020). Similar to our finding, a previous report also showed that IRF4 expression is necessary for Epstein-Barr virus (EBV)-mediated lymphoblastoid B cell transformation (Ma et al., 2017), and the IRF4 superenhancer region is an EBV-regulated super-enhancer, which is essential for transformation of resting B lymphocytes to continuously proliferating lymphoblastoid cell line (Jiang et al., 2017).

KSHV has evolved to maintain a mysterious lncRNA, PAN RNA, which is expressed at a very high copy number in the presence of ORF57 protein (Song et al., 2001), and deletion of PAN RNA or ORF57 significantly impairs the entire viral lytic gene expression program (Campbell et al., 2018; Majerciak et al., 2007; Rossetto et al., 2013). How PAN RNA expression influences the activation of distantly localized viral gene promoters was a remaining question. This study suggests that CHD4 sequestration from the KSHV genome by PAN RNA might be one of the mechanisms to release KSHV from CHD4-mediated gene repression. Similar to CHD4, we previously demonstrated that PAN RNA expression sequesters LANA from unique regions of KSHV genomes (Campbell et al., 2014). Our CUT&RUN studies showed that CHD4 and LANA colocalized on both the KSHV and host genomes and they could physically interact with each other; thus, we favor the idea that reactivation is triggered by detachment of LANA/CHD4 complex-loaded TR fragments from the KSHV unique region by robust expression of lncRNA, whose expression is directly activated by K-Rta, and also enhanced by ORF57. However, further studies will be required to fully understand the mechanism of KSHV episome displacement and determine if detachment of KSHV episome from CHD4-enriched host chromosomes also plays a role in KSHV inducible lytic gene expression.

In summary, we have demonstrated that CHD4 is a key regulator of the KSHV latency-lytic switch. With strong effects of CHD4 on KSHV replication, it will be important to study how the CHD4/ADNP/LANA complex is regulated in infected cells, and also an association with KSHV-mediated disease progression in individuals who unfortunately possess CHD4 mutations.

### Limitations of the study

One of the limitations of this study is use of cell lines and have not validated the findings in clinical samples. We understand those PEL lines are transformed cells, which may not accurately represent natural KSHV infection. In addition, our genomic studies were performed with cell population. Accordingly, what we see as peaks for protein binding or episome tethering sites are major binding sites from the average of given cells. Individual cells also contain 50 to 100 of KSHV episomes. Thus, our experimental setting is likely to bury minor protein binding sites and also episome tethering regions in unique cell populations, which may play very important role in the KSHV life cycle.

## STAR★METHODS

Detailed methods are provided in the online version of this paper and include the following:

### RESOURCE AVAILABILITY

#### Lead contact

Further information and requests for resources and reagents should be directed to and will be fulfilled by the lead contact, Yoshihiro Izumiya (yizumiya@ucdavis.edu).

#### Materials availability

All unique reagents generated in this study are available upon reasonable request from the Lead contact, Yoshihiro Izumiya (yizumiya@ucdavis.edu).

#### Data and code availability

- CUT&RUN and CHI-C sequencing data have been deposited at GEO and are publicly available as of the date of publication. Accession numbers are listed in the Key resource table. Microscopy data reported in this paper will be shared by the Lead contact upon request.
- This paper does not report original code.
- Any additional information required to reanalyze the data reported in this paper is available from the Lead contact upon request.

### EXPERIMENTAL MODEL AND SUBJECT DETAILS

#### Cell culture

*i*SLK.219 cells were maintained in DMEM supplemented with 10% FBS, 1% penicillin-streptomycin-L-glutamine solution, 10 μg/mL puromycin, 400 μg/mL hygromycin B, and 250 μg/mL G418. *i*SLK cells were obtained from Dr. Don Ganem (Novartis Institute for Biomedical Research) and were maintained in DMEM supplemented with 10% FBS, 1% penicillin-streptomycin-L-glutamine solution and 10 μg/mL puromycin. BC-1 and BC-3 cell lines were obtained from ATCC (Manassas, VA, USA), expanded to obtain early passage stocks, and stored. The BCBL-1 cell line was also obtained from Dr. Ganem (University of California San Francisco). BC-1, BC-3, and BCBL-1 cell lines were cultured in RPMI 1640 medium supplemented with 15% FBS and 1% penicillin-streptomycin-L-glutamine solution. TREx BCBL-1 cells, which can induce Flagx3-HAx3-K-Rta in tetracycline inducible manner, was prepared before (Campbell et al., 2018) were maintained in RPMI 1640 medium supplemented with 10% FBS, 1% penicillin-streptomycin-L-glutamine, 250 μg/mL hygromycin B, and 100 μg/mL blasticidin.

#### Recombinant KSHV

##### Construction of mini-Turbo KSHV BAC16

Recombinant KSHV was prepared by following a protocol for En passant mutagenesis with a two-step markerless red recombination technique (Tischer et al., 2010). Briefly, the codon optimized mini-TurboID coding sequence (Kumar et al., 2021), which also encodes the 3x Flag tag, was first cloned into a pBS SK vector (Thermo Fisher, Waltham, MA USA). The pEPkan-S plasmid was used as a source of the kanamycin cassette, which includes the I-SceI restriction enzyme site at the 5′-end of the kanamycin coding region (Tischer et al., 2010). The kanamycin cassette was amplified with primer pairs with homology arms and cloned into the mini-TurboID coding region at a unique restriction enzyme site. The resulting plasmid was used as a template for another round of PCR to prepare a transfer DNA fragment for markerless recombination with BAC16 (Brulois et al., 2012). Recombinant BAC16 clones with insertion and also a deletion of the kanamycin cassette in the BAC16 genome were confirmed by colony PCR with appropriate primer pairs. Recombination junctions and adjacent genomic regions were amplified by PCR and the resulting PCR products were directly sequenced with the same primers to confirm in-frame insertion of the mini-TurboID cassette into the BAC16 DNA for mini-TurboID-ORF57 and -LANA. Insertion of PAN RNA MRE mutations was performed by using primer pairs that encode the intended mutations. Primer pairs used to amplify the kanamycin cassette and recombination, deletion of the kanamycin cassette, and confirmation of mutations were performed as described above. The resulting recombinant BAC16 was confirmed by restriction enzyme digestions (*Hind*III and *Bgl*II) to determine if there were any large DNA deletions. Two independent BAC16 clones were generated for each mini-TurboID tagged recombinant KSHV virus as biological replicates and one of the clones was used for protein ID. Entire BAC16 DNAs were subsequently fully sequenced on an Illumina MiSeq System.

### METHOD DETAILS

#### Capture Hi-C

KSHV Capture Hi-C (CHi-C) was performed using a robust *in situ* CHi-C protocol with kitted reagents from Arima Genomics (San Diego, CA, USA) based on methods described for *in situ* Hi-C (Belaghzal et al., 2017; Rao et al., 2014), CHi-C (Jung et al., 2019; Schmitt et al., 2016; Schoenfelder et al., 2018), and as described previously (Campbell et al., 2018). Briefly, cells were crosslinked with 2% formaldehyde, lysed, and the genomic DNA digested with a cocktail of 4-cutter restriction endonucleases by incubation for 30 minutes at 37°C. The 5′-overhangs were then filled in and labeled with biotinylated dATP (biotin-14-dATP) by incorporation with Klenow fragment of DNA polymerase I (incubation for 45 minutes at 25°C). Ligation of the spatially proximal blunt-ended fragments was then performed with T4 DNA ligase (incubation for 15 minutes at 25°C). The formaldehyde crosslinks were reversed and the proximally-ligated, chimeric DNA products were purified with Agencourt AMPure XP paramagnetic beads (Beckman Coulter, Brea, CA, USA). The DNA was then fragmented to an average size of 400 bp with a Covaris E220 Focused-ultrasonicator (Covaris, Inc., Woburn, MA, USA) and size-selected to have a fragment size distribution of 200–600 bp with AMPure XP beads. The biotinlabeled ligation products were then selectively enriched by affinity capture with streptavidin magnetic beads (Arima Enrichment Beads). Subsequently, sequencing libraries were prepared with the Kapa HyperPrep Kit with Library Amplification Module (Roche, Basel, Switzerland) using a modified protocol for on-bead end repair, dA-tailing, and ligation of Illumina TruSeq sequencing adaptors.

The KSHV CHi-C library was then prepared from the Hi-C libraries as previously described (Campbell et al., 2018). Briefly, target enrichment for KSHV genomic content was performed by solution hybridization with a custom-designed KSHV genomic capture probe library (xGen Lockdown Probes; Integrated DNA Technologies, Inc., Coralville, IA) (Campbell et al., 2018) and subsequent capture of the hybridized targets with streptavidin beads (DynaBeads MyOne Streptavidin C1; Thermo Fisher) according to the manufacturer’s standard protocol (Integrated DNA Technologies, Inc., Coralville, IA). The KSHV genome-enriched CHi-C library DNA was eluted and PCR enrichment (12 cycles) performed with high-fidelity KAPA HiFi HotStart DNA Polymerase (Kapa Biosystems, Inc., Wilmington, MA). Libraries were multiplex sequenced (2 × 150bp, paired-end, ~50 million mapped reads/mate pairs per sample) on an Illumina Hiseq 4000 sequencing system.

#### Cleavage Under Targets and Release Using Nuclease (CUT&RUN)

CUT&RUN (Skene and Henikoff, 2017) was performed essentially by following the online protocol established by Dr. Henikoff’s lab with a few modifications. Cells were washed with PBS and wash buffer [(20 mM HEPES-KOH pH 7.5, 150 mM NaCl, 0.5 mM Spermidine (Sigma, S2626) and proteinase inhibitor (Roche)]. After removing the wash buffer, cells were captured on magnetic ConA beads (Polysciences, PA, USA) in the presence of CaCl_2_. Beads/cells complexes were washed 3 times with digitonin wash buffer (0.02% digitonin, 20 mM HEPES-KOH pH 7.5, 150 mM NaCl, 0.5 mM Spermidine and 1x proteinase inhibitor), aliquoted, and incubated with specific antibodies in 250 μL volume. The antibodies used in this study were: rabbit monoclonal anti-CHD4 (Cell Signaling, D4B7; 1:50); rat monoclonal anti-LANA (Millipore-Sigma, clone LN53; 1:50); rabbit anti-ADNP polyclonal (Invitrogen, PA5-52286; 1:100); rabbit monoclonal anti-Acetylated Histone H3 lysine 27 (Cell Signaling, D5E4; 1:100), rabbit monoclonal anti-H3 monomethyl lysine 4 (Cell Signaling, D1A9; 1:100), and rabbit monoclonal anti-H3 tri-methyl lysine 27 (Cell Signaling, C36B11; 1:100). After incubation, unbound antibody was removed by washing with digitonin wash buffer 3 times. Beads were then incubated with recombinant pAG-MNase, which was purified from *E.coli*, in 250 μL digitonin wash buffer at 1.0 μg/mL final concentration for one hour at 4°C with rotation. Unbound pAG-MNase was removed by washing with digitonin wash buffer 3 times. Pre-chilled 2 mM CaCl_2_ containing digitonin wash buffer (200 μL) was added to beads and incubated on ice for 30 min. The pAG-MNase digestion was halted by the addition of 200 μL 2× STOP solution (340 mM NaCl, 20 mM EDTA, 4 mM EGTA, 50 μg/mL RNase A, 50 μg/mL glycogen). The beads were incubated with shaking at 37°C for 10 min in a tube shaker at 500 rpm to release digested DNA fragments from the insoluble nuclear chromatin. The supernatant was collected after centrifugation (16,000 × g for 5 min at 4°C) and placed on a magnetic stand. DNA was extracted using the NucleoSpin kit (Takara Bio, Kusatsu, Shiga, Japan). Sequencing libraries were then prepared from 3 ng of CUT&RUN DNA with the Kapa HyperPrep Kit (Roche) according to the manufacturer’s standard protocol. Libraries were multiplex sequenced (2 × 150bp, paired-end) on an Illumina HiSeq 4000 sequencing system to yield ~15 million mapped reads per sample. When necessary, *E. coli* genomic DNA read from a pAG-MNase incubation were used to normalize data as described previously (Skene and Henikoff, 2017).

#### Genomic data analysis

The HiC-pro 2.11.1 pipeline (Servant et al., 2015) was used to align sequences from the Capture Hi-C experiments against a combined assembly of reference genomes; the human hg19 (GRCh37) and KSHV (NC_009333.1). The reads were filtered for only uniquely mapped reads pairs by identifying the intersection of each read-end, and the valid reads were filtered for by removing reads corresponding to self-circle, dangling-end, error, extra dangling-end, too short, too large, duplicated, and/or random breaks. The valid read pairs were stored as matrices and binned with a resolution of 10,000 bp. Iterative Correction and Eigenvector decomposition (ICE) normalization was used to treat the data with default parameters. The normalized counts were filtered to keep only the counts that mapped to both the human and KSHV genome by using Python with Pandas library. The results were visualized as dot plots using Matplotlib library.

The normalized counts were also used as input in subsequent analyses. In the analysis of KSHV episome localization near centromeres, centromeres of human chromosome (hg19) were downloaded from the UCSC Table Browser with a filter of ‘centromere’ as gap type. For each chromosome, the sum of all counts was compared to both the sums of the counts in the regions near the centromere at the 5′ and −3′ sides, both of which are 1% the size of the chromosome (the values were first normalized as per 100,000 bp before the comparisons). The similarity of CHi-C chimeric sequence reads among BC-1, BC-3, and BCBL-1 were performed as follows. Genomic regions with normalized counts >0 were extracted and then the intersect and union regions were determined using Intervene v0.6.4 (Khan and Mathelier, 2017). The similarity percentage was calculated based on the Jaccard similarity index.

FASTQ files for the capture Hi-C experiments were processed through the HiCUP (v0.7.4) pipeline (Wingett et al., 2015) using a combined human hg19 (GRCh37) and KSHV (NC_009333.1) genome. Valid interaction products called by HiCUP were converted into Juicebox (Durand et al., 2016) input format (.hic file), which stores the normalized and un-normalized contact matrices as a highly compressed binary file, by using a series a scripts provided by HiCUP (hicup2homer) and HOMER (makeTagDirectory and tagDir2-hicFile) (Heinz et al., 2010). Juicebox was utilized to facilitate adjustments of resolution and normalization, intensity scaling, zooming, and addition of annotation tracks.

CUT&RUN sequence reads were aligned to the human hg38 reference genome and reference KSHV genome sequence (Human herpesvirus 8 strain: GQ994935.1) with Bowtie2 v2.3.5.1 (Langmead and Salzberg, 2012) and/or HISAT2 v2.1.0 (Kim et al., 2019). MACS2 (Model-based Analysis of ChIP-Seq) v2.1.1.1.20160309 was used for detecting peaks (Zhang et al., 2008) following the developer’s manual. Peaks and read alignments were visualized using the Integrated Genome Browser (IGB) (Freese et al., 2016). Heatmaps and average profile plots were drawn from bed files created by MACS and bam files using R package, ngsplot v2.63 (Shen et al., 2014). After subtracting the minimum value of average profile as background signal, relative average profile plot was drawn. Accumulation index was calculated as proportion of area under the curve.

#### Single cell RNA sequencing data analysis

Single cell data was analyzed with the Cell Ranger v2.1 pipeline (10x Genomics). The pipeline included alignment to the hg38 human reference genome and human herpesvirus 8 strain (GQ994935.1) reference genome, t-distributed stochastic neighbor embedding (tSNE), and K-means clustering. Read count matrices obtained from the pipeline were normalized using log normalization method with “Seurat” R package (Stuart et al., 2019). To perform correlation analysis, the expression of the KSHV genes was summarized, log transformed and resulting values divided by 14 equal intervals to have more than 50 cells in each interval. The median value of gene expression was calculated for the cells in each interval. The median values were used to analyze and visualize the correlation of host cellular and viral gene expression.

#### Immunofluorescence staining analyses

*i*SLK cells latently infected with BAC16-Wt were seeded onto glass coverslips and transfected with pLenti-Flag-mCHD4-myc WT or pLenti-Flag-mCHD4-myc mutant using Lipofectamine 2000 reagent according to the manufacturer’s protocol. pLenti-Flag-mCHD4-myc was obtained from a commercial source (Origene, MD, USA). Point mutations were inserted by synthesizing DNA fragments that contain intended mutations and replaced at SexAI-AgeI restriction sites with In-FusionHD (Takara Bio, Japan). At 24 hrs after transfection, 1 μg/mL of doxycycline and 20 ng/mL of 12-O-Tetradecanoylphorbol-13-acetate (TPA) were added, and cells were cultured for a further 26 hrs in the presence of doxycycline and TPA. The cells were then fixed with 4% paraformaldehyde, permeabilized with 0.2% Triton X-100, and labeled with anti-RNAPlI mouse monoclonal antibody (Millipore-Sigma, clone CTD4H8, 1:100) and anti-FLAG rabbit polyclonal antibody (Sigma, F7425, 1:100), followed by Alexa 555-anti mouse IgG and Alexa 647-anti rabbit IgG (Thermo Fisher). Nuclei were counterstained with 1μg/ml of Hoechst 33342 (Thermo Fisher). The labeled cells were observed with a Keyence BZ-X710 fluorescence microscope (Keyence, Osaka, Japan) with standard DAPI, GFP, TRITC and Cy5 filter sets (Chroma Technologies, Bellows Falls, VT, USA).

#### DNA-FISH combining with LANA immunostaining

BCBL-1 cells were washed with PBS twice and spotted on coverslips and fixed with 4% formaldehyde-PBS for 10 min at RT. Cells were subsequently incubated with PBS with 100 mM glycine to quench residual formaldehyde. Cells were treated with 0.1% Triton-X and 0.05% SDS with RNaseA for 15 min at 37°C. Coverslips were washed with PBS twice, 70% ethanol once, 85% ethanol once, 100% ethanol once, and dried completely. Cy5-conjugated CENPB-Cy5 PNA probe was obtained from a commercial source (PNA Bio, Thousand Oaks, CA, USA) and coverslips were incubated with hybridization buffer (200 nM PNA probe, 2xSSC, 20% Dextran, 1 mg/mL yeast tRNA, 60% formamide) on a glass slide on a heating plate at 57°C for 10 min and then 37°C for another 30 min. Utilization of PNA probes avoids harsh conditions often used for a larger DNA hybridization, which we found to produce unacceptable noise and not readily compatible with subsequent protein staining. Coverslips were placed in 6-well plates and washed 3 times with warmed 0.1% Tween 20 in PBS (57°C) and PBS once. Primary antibody was incubated for LANA staining in PBS with 1 mg/mL yeast tRNA. After washing with PBS 3 times, secondary antibody (Alexa 488-anti Rat IgG) was incubated in PBS with yeast tRNA for 1 h at 37°C. After washing 3 times with PBS, the coverslips were mounted with antifade reagent (Thermo Fisher), and images were captured with a Keyence microscope.

#### 3D fluorescence microscopy and quantitative image analysis

Widefield 3D fluorescence imaging was performed on a Keyence BZ-X710 fluorescence microscope (Keyence, Osaka, Japan) equipped with live-cell environmental chamber. Cells fluorescently labeled as previously described were observed with standard DAPI, GFP, TRITC and Cy5 filter sets (Chroma Technologies, Bellows Falls, VT, USA). High resolution image stacks were acquired in each color channel using Keyence onboard software. Image stacks were converted to greyscale TIF images that were uniformly corrected for haze reduction, deblurring and contrast enhancement.

Image data were subsequently exported to the Volocity® Multi-Dimensional Imaging Platform (Quorum Technologies Inc., Ontario, Canada) for 3D visualization and quantitative analysis. For image-based cytometry measurements, individual cells within a given field of view were automatically identified and fluorescent objects and/or region of interest in the various color channels were quantified and tabulated. For qualitative assessment of LANA and centromere oligo probes for DNA-FISH, 3D image stacks were similarly compiled and displayed in 3D opacity mode using Volocity Visualization toolset. Reconstructed 3D images were used for distance-measurement analysis: briefly, fluorescent LANA dots (green) and DNA centromeres (purple) in individual cell nuclei were automatically identified in Volocity Quantitation. The minimum distance from each centromere to the nearest LANA dot could then be extracted from the statistical measurements and displayed as histograms for subsequent analysis.

#### Lentivirus production and transduction

For CHD4 knock-down experiments, the following shRNA sequences from Sigma pLKO.1 shRNA libraries were used: shRNA#1: CCTTACTAGAATTGGTGTTAT; shRNA#2: GCTGACACAGTTATTATCTAT. Lentiviruses generated from the CHD4-expressing lentivector, CHD4-targeting shRNAs, and non-targeting scramble shRNA (Addgene, #1864) were produced in 293T cells. The vectors were co-transfected with psPAX2 (Addgene, #12260) and pMD2.G (Addgene, #12259) into 293T cells using polyethylenimine (PEI). Supernatants were collected 48 and 72 hrs post-transfection. Cells were infected with lentiviruses in the presence of 8 μg/mL polybrene and subsequently incubated for 72 h to allow for protein expression or knock-down. To generate CHD4 knock-down cell lines, 293T cells were infected with lentivirus for 24 h. Cells were then cultured in selection medium containing 1 μg/mL puromycin for two weeks to obtain stable CHD4 knock-down cell lines.

#### Preparation of purified KSHV and de novo infection

*i*SLK.219 cells latently infected with recombinant KSHV were cultured in eight to ten 150 mm culture dishes until 80% confluent. For reactivation, cultures were re-fed with complete DMEM (without selection drugs) containing 0.3 mM sodium butyrate and 1 μg/mL doxycycline. The cells were further cultured for 5 days in the presence of sodium butyrate and doxycycline. The culture supernatant was centrifuged at 300 × g for 10 min, and then passed through a 0.8-μm filter to remove cellular debris, and then viral particles were concentrated by ultracentrifugation at 25,000 rpm for 2 hrs at 4°C with a Beckman SW28 rotor. The viral precipitates were resuspended in 500 μL of DMEM along with the residual ~500 μL media in the centrifuge tube and stored at −80°C until use. For *de novo* infection, CHD4-KD 293T cells were seeded at 4 × 10^5^ cells/well in 6-well plates. After overnight culture, viral stocks were added to the cultures and the infection was allowed to proceed for 24 hrs. Cells were monitored for GFP expression and RNA was purified using the RNeasy Mini Kit (Qiagen, Venlo, Netherlands).

#### Flow cytometry

293T cells were transfected with 10 pmol CHD4 siRNA using Lipofectamine RNAiMax reagent (Thermo Fisher) for 48 hrs. Cells were infected with r.219 virus for 96 hrs in DMEM media containing 5% FBS. After that, cells were trypsinized and washed with PBS two times followed by fixation with 2% PFA containing 1 mM EDTA for 10 minutes at 37 °C. Cells were then washed with PBS twice and resuspended in PBS containing 1 mM EDTA. Cells were passed through a 0.45-μm strainer to obtain single cells. A BD “Fortessa” cytometer was used for FACS analysis, and FlowJo software (Tree Star) was used for data analysis.

#### Purification of recombinant protein

*Spodoptera frugiperda* Sf9 cells (Millipore) were maintained in Ex-Cell 420 medium (Sigma), and recombinant baculoviruses were generated with the Bac-to-Bac baculovirus expression system as previously described (Izumiya et al., 2009; Kim et al., 2013). Transfer plasmid, pFAST-BAC1 vector was modified by inserting a Flag tag at the N-terminus, and CHD4, ORF57, p65, and Luciferase cDNAs were cloned into the CpoI (*RsrII*) site. The cDNA of ADNP, which also include C-terminal His tag was synthesized and cloned into *BamHI* and *PstI* restriction enzyme sites by HiFi Gene assembly (NEB). Recombinant baculovirus bacmid DNA was transfected into Sf9 cells by using polyethylenimine (Sigma), and recombinant viruses were subsequently amplified twice. Expression of recombinant proteins was confirmed by immunoblotting with anti-Flag monoclonal antibody (Sigma) or anti-His (BioRad). Largescale cultures of Sf9 cells (50 mL) were infected with recombinant baculovirus at a multiplicity of infection (MOI) of 0.1–1.0, and cells were harvested 48 hrs after infection. Recombinant proteins were purified as described previously (Izumiya et al., 2005). The purity and amount of protein were measured by SDS-PAGE and Coomassie blue staining, using bovine serum albumin (BSA) as a standard.

#### *In vitro* interaction assays

Baculoviruses expressing Flag-CHD4, His-ADNP, or Flag-LANA were co-infected in SF9 cells and purified by Flag tag capture in the presence of 500 mM NaCl and 10% glycerol. Purified complex was re-suspended in the binding buffer (20 mM HEPES [pH 7.9], 150 mM NaCl, 1 mM EDTA, 4 mM MgCl_2_, 1 mM dithiothreitol, 0.02% NP-40, 10% glycerol supplemented with 1 mg/mL BSA, and 13 protease inhibitor cocktail) and the antibody was incubated at 1:100 dilution for 1 h at 4°C to form an immunocomplex. The immunocomplex was captured with 10 μL of protein A/G magnetic beads mixture. Beads were washed three times with binding buffer and subjected to SDS-PAGE after eluting proteins in sample buffer. The interaction was probed by immunoblotting with anti-CHD4, anti-ADNP, or anti-LANA antibody.

For RNA or DNA pull-down, a final concentration of 100 nM of each biotinylated RNA fragment (e.g. 1.3 μg for full length PAN RNA transcribed *in vitro* or 2.6 μg of dsDNA generated by PCR with 5′-biotinylated primer) and purified proteins (100 nM in final) was incubated in RNA-binding buffer (50 mM HEPES [pH 7.9], 150 mM KCl, 5% glycerol, 0.02% NP-40, 100 μg/mL yeast tRNA, 2 mM MgCl_2_) supplemented with RNAse inhibitor in 40 μL reaction for 1 h at 4°C. Biotinylated RNA or dsDNA was captured with streptavidin conjugated magnetic beads, washed with binding buffer 3 times, and proteins were eluted in sample buffer. Precipitated proteins were visualized by immunoblotting with either anti-Flag antibody (Sigma) or specific antibody as indicated in figure legends.

#### Quantification of viral copy number

Two hundred microliters of cell culture supernatant were treated with DNase I (12 μg/mL) for 15 min at room temperature to degrade unencapsidated DNA. This reaction was stopped by the addition of EDTA to 5 mM followed by heating at 70°C for 15 min. Viral genomic DNA was purified using the QIAamp DNA Mini Kit according to the manufacturer’s protocol and eluted in 100 μL of buffer AE. Four microliters of the eluate were used for real-time qPCR to determine viral copy number, as described previously (Izumiya et al., 2009).

#### Real-time RT-PCR

Total RNA was isolated using the Quick-RNA miniprep kit (Zymo Research, Irvine, CA, USA). First-strand cDNA was synthesized using the High Capacity cDNA Reverse Transcription Kit (Thermo Fisher, Waltham, MA USA). Gene expression was analyzed by real-time qPCR using specific primers for KSHV ORFs designed by Fakhari and Dittmer (Fakhari and Dittmer, 2002). We used 18S ribosomal RNA or GAPDH as an internal standard to normalize viral gene expression.

#### Western blotting

Cells were lysed in IP lysis buffer (25 mM Tris-HCl pH 7.4, 150 mM NaCl, 1% NP-40, 1 mM EDTA, 5% glycerol) containing protease inhibitors (Roche, Basel, Switzerland). Total cell lysates (25 μg) were boiled in SDS-PAGE loading buffer and subjected to SDS-PAGE and subsequently transferred to a polyvinylidene fluoride membrane (Millipore-Sigma, St. Louis, MO, USA) using a semidry transfer apparatus (Bio-Rad, Hercules, CA, USA). The streptavidin-HRP conjugate was used at 1:3000 dilution. Final dilutions of the primary antibodies were 1:3,000 for anti-Flag mouse antibody, 1:1,000 for anti-LANA rat antibody, anti-CHD4 rabbit, and anti-ADNP rabbit antibodies. Membrane washes and secondary antibody incubations were performed as described previously (Izumiya et al., 2009).

#### Affinity purification of biotinylated proteins

KSHV LANA-mTID cells were incubated with 100 μM D-biotin for 60 mins at 37°C. To stop the reaction, cells were incubated at 4°C for 60 mins. Cells were washed with cold PBS three times followed by lysis with SUMO lysis buffer (Tris-Cl PH 6.8, 1% SDS and 10% glycerol). Lysates were immediately boiled for 10 min, and protein was quantified by Bradford assay (Bio-Rad). The biotinylation signal was confirmed by western blotting using Strep-HRP.

Affinity purification was performed with streptavidin-coated magnetic beads (Thermo-Fisher). Briefly, 150 μL magnetic beads/sample were pre-washed with RIPA lysis buffer (150 mM NaCl, 5 mM EDTA (pH 8), 50 mM Tris (pH 8), 1% NP-40, 0.5% sodium deoxycholate, 0.1% SDS) 3 times. A total of 3 mg of whole cell lysate was incubated with pre-washed streptavidin beads after diluting with RIPA buffer for 10 times, at room temperature for 1h with rotation. The beads were collected using a magnetic stand and washed three times with wash buffer according to the manufacturer’s protocol. Finally, beads were resuspended in 200 μL of wash buffer and sent to the UC Davis Proteomics core for on bead digestion and LC-MS/MS analysis.

#### MS sample preparation

Protein samples on magnetic beads were washed four times with 200 μL of 50 mM ammonium bicarbonate (AMBIC) with a twenty-minute shake time at 4°C in between each wash. Roughly 2.5 μg of trypsin was added to the beads and AMBIC and the samples were digested overnight at 800 rpm shake speed. After overnight digestion, the supernatant was removed, and the beads were washed once with enough 50 mM AMBIC to cover them. After 20 minutes at a gentle shake the wash was removed and combined with the initial supernatant. The peptide extracts were reduced in volume by vacuum centrifugation and a small portion of the extract was used for fluorometric peptide quantification (Thermo Scientific Pierce). One microgram of sample based on the fluorometric peptide assay was loaded for each LC-MS analysis.

Digested peptides were analyzed by LC-MS/MS on a Thermo Scientific Q Exactive Orbitrap Mass spectrometer in conjunction with Proxeon Easy-nLC II HPLC (Thermo Scientific) and Proxeon nanospray source. The digested peptides were loaded on a 100 micron × 25 mm Magic C18 100Å 5U reverse phase trap where they were desalted online before being separated using a 75-micron × 150 mm Magic C18 200Å 3U reverse-phase column. Peptides were eluted using a 60-minute gradient with a flow rate of 300 μL/min. An MS survey scan was obtained for the m/z range 300–1600, MS/MS spectra were acquired using a top 15 method, where the top 15 ions in the MS spectra were subjected to HCD (High Energy Collisional Dissociation). An isolation mass window of 2.0 m/z was used for the precursor ion selection, and normalized collision energy of 27% was used for fragmentation. A fifteen second duration was used for the dynamic exclusion.

#### MS/MS analysis

Tandem mass spectra were extracted and charge state deconvoluted by Proteome Discoverer (Thermo Scientific). All MS/MS samples were analyzed using X! Tandem (The GPM, thegpm.org; version X! Tandem Alanine (2017.2.1.4)). X! Tandem was set up to search the Human and Kaposi’s Sarcoma Herpes virus database (149182 entries) assuming the digestion enzyme trypsin. X! Tandem was searched with a fragment ion mass tolerance of 20 PPM and a parent ion tolerance of 20 PPM. Carbamidomethyl of cysteine and selenocysteine was specified in X! Tandem as a fixed modification. Glu->pyro-Glu of the N-terminus, ammonia-loss of the N-terminus, gln->pyro-Glu of the N-terminus, deamination of asparagine and glutamine, oxidation of methionine and tryptophan and dioxidation of methionine and tryptophan were specified in X! Tandem as variable modifications.

Scaffold (version Scaffold_4.8.4, Proteome Software Inc., Portland, OR) was used to validate MS/MS-based peptide and protein identifications. Peptide identifications were accepted if they could be established at greater than 98.0% probability by the Scaffold Local FDR algorithm. Peptide identifications were also required to exceed specific database search engine thresholds. Protein identifications were accepted if they could be established at greater than 5.0% probability to achieve an FDR less than 5.0% and contained at least 2 identified peptides. Protein probabilities were assigned by the Protein Prophet algorithm (Nesvizhskii et al., 2003). Proteins that contained similar peptides and could not be differentiated based on MS/MS analysis alone were grouped to satisfy the principles of parsimony. Proteins sharing significant peptide evidence were grouped into clusters.

#### Pathway analysis

The proteins identified to be interacting with ORF57 were used for gene ontology analysis. The top gene ontology processes were enriched by the Metascape web-based platform, and the Metascape software was used for gene ontology analysis (Zhou et al., 2019).

### QUANTIFICATION AND STATISTICAL ANALYSIS

Information on specific quantification methods is described in associated Method details, or Figure legends. Results are shown as mean ± SD from at least three independent experiments. GraphPad Prism 8.0.1 software or Microsoft Excel was used to calculate the statistical analysis. Data were analyzed using unpaired Student’s t test, or ANOVA followed by Tukey’s HSD test. A value of p < 0.05 was considered statistically significant.

## Supplementary Material

1

2

## Figures and Tables

**KEY RESOURCES TABLE:** 

REAGENT or RESOURCE	SOURCE	IDENTIFIER

Antibodies		

Anti-HHV-8 LNA-1 Antibody, clone LN53	Sigma-Aldrich	Cat# MABE1109
CHD4 (D4B7) Rabbit mAb	Cell Signaling Technology	Cat# 12011S; RRID: AB_2734702
ADNP Polyclonal Antibody	Thermo Fisher	Cat# PA5-52286; RRID: AB_2637725
Monoclonal ANTI-FLAG® M2 antibody produced in mouse	Sigma-Aldrich	Cat# F1804; RRID: AB_262044
Acetyl-Histone H3 (Lys27) (D5E4) XP® Rabbit mAb	Cell Signaling Technology	Cat# 8173; RRID: AB_10949503
Tri-Methyl-Histone H3 (Lys27) (C36B11) Rabbit mAb	Cell Signaling Technology	Cat# 9733; RRID: AB_2616029
Mono-Methyl-Histone H3 (Lys4) (D1A9) XP® Rabbit mAb	Cell Signaling Technology	Cat# 5326; RRID: AB_10695148
Anti-RNA polymerase II Antibody, clone CTD4H8	Sigma-Aldrich	Cat# 05-623; RRID:AB_309852
Anti-mouse IgG, HRP-linked Antibody	Cell Signaling Technology	Cat# 7076; RRID: AB_330924
Anti-rabbit IgG, HRP-linked Antibody	Cell Signaling Technology	Cat# 7074; RRID: AB_2099233
Anti-β-Actin antibody, Mouse monoclonal	Sigma-Aldrich	Cat# A1978; RRID: AB_476692
normal mouse IgG	Santa Cruz Biotechnology	Cat# sc-2025; RRID: AB_737182

Bacterial and virus strains		

KSHV virions from iSLK.219	In this study	N/A
KSHV virions from iSLK LANA-mTID	In this study	N/A
KSHV virions from iSLK.BAC16	In this study	N/A

Chemicals, peptides, and recombinant proteins		

Lipofectamine RNAiMAX	Invitrogen	Cat# 13778150
Lipofectamine 2000	Invitrogen	Cat# 11668019
Protease inhibitor cocktail	Sigma-Aldrich	Cat# 11697498001
D-Biotin	Invitrogen	Cat# B1595
Pierce™ Streptavidin Magnetic Beads	Thermo Scientific	Cat# 88816
Streptavidin, horseradish peroxidase conjugate	Invitrogen	Cat# S911
Hygromycin B	Enzo	Cat# ALX-380-306-G001
Puromycin	InvivoGen	Cat# ant-pr-1
G418	InvivoGen	Cat# ant-gn-1
Blasticidin	InvivoGen	Cat# ant-bl-05
DMEM, high glucose	Gibco	Cat# 11965092
RPMI 1640 Medium	Gibco	Cat# 61870036
FBS	Gibco	Cat# 26140-079
Opti-MEM™ I Reduced Serum Medium	Gibco	Cat# 31985062
Penicillin-Streptomycin-Glutamine (100X)	Gibco	Cat# 10378016
Trypsin-EDTA (0.5%)	Gibco	Cat# 15400054
CHD4 protein	In this study	N/A
LANA protein	In this study	N/A
ADNP protein	In this study	N/A
GST protein	In this study	N/A
GST LANA 4–150, 50–200, 100–250, 150–330, 870–1020, 870–1070, 1020–1162	In this study	N/A
Luciferase protein	In this study	N/A
Spermidine	Sigma-Aldrich	Cat# S2626
BioMag®Plus Concanavalin A	Polysciences	Cat# 86057-10
Digitonin	Spectrum	Cat# DI120
pAG-MNase	In this study	N/A
RNAase A	Macherey-Nagel	Cat# 740505
AMPure XP paramagnetic beads	Beckman	Cat# A63880

Critical commercial assays		

Arima-HiC kit	Arima Genomics	N/A
HiScribe™ T7 High Yield RNA Synthesis Kit	NEB	Cat# E2040S
NEBuilder® HiFi DNA Assembly Master Mix	NEB	Cat# E2621L

Deposited data		

CHi-C	In this study	GEO: GSE163694
CUT&RUN	In this study	GEO: GSE163098

Experimental models: Cell lines		

Human: iSLK.219	Myoung and Ganem, 2011	N/A
Human: TREx BCBL1	Nakamura et al., 2003	N/A
Human: 293FT	Invitrogen	Cat# R70007
Human: BC1	ATCC	CRL-2230
Human: BC3	ATCC	CRL-2277
Human: BCBL1	Renne et al., 1998	N/A
Human: iSLK LANA-mTID	In this paper	N/A
Human: iSLK	Myoung and Ganem, 2011	N/A
Human: iSLK mTID-57 PAN Wt	In this paper	N/A
Human: iSLK mTID-57 PAN Mt	In this paper	N/A

Oligonucleotides		

Primer for qPCR	In this paper	Table S3
ORF57 TurboID FW Recombination: GCTGCCAGGTTCCCAAAATAGCCC GCGGCATACGGCTCACTTCCCCCC ACATTCCCCCCCGTGCACAATATAA GAACCAAAGGACATGGATTATAAGG ATGATGACAAGGGGGAC	In this paper	N/A
ORF57 TurboID RV Recombination: AGAAATGGGAAAAGTCTGTTGTAG ACGAGGACTTACCCTCTAGGATGC CCTTCATAATGTCCATGTCTATCAT TGCTTGTACGTCCGCTCGGCTAAA CTCCTTCTCTGCACTTC	In this paper	N/A
LANA Turbo ID FW Recombination: CCTTGTGGTCACTACGGGTATTGC ATAATGTGAATATACTGCCACCGC CTCCATAATTTTACTTTGGTTGTCA GACCAGATTTCCCGAGGATGGATT ATAAGGATGATGACAAGGGGGAC	In this paper	N/A
LANA TurboID RV Recombination:		
CTGTTTCGTTTCCTACAACTTCCTC TCGTTAAGGGCGCGCCGGTGCTC CGTCCCGACCTCAGGCGCATTCC CGGGGGCGCGTCCGCTCGGCTA AACTCCTTCTCTGCACTTCTGAGA	In this paper	N/A
siRNA targeting sequence	In this paper	Table S4
*In vitro* PAN RNA interaction assay	In this paper	Table S5
Custom-designed KSHV genomic capture probe library	IDT	N/A

Recombinant DNA		

CHD4 (NM_001273) Human Tagged ORF Clone	Origene	Cat# RC224232L1
Chd4 (NM_145979) Mouse Tagged ORF Clone	Origene	Cat# MR212098

Software and algorithms		

GraphPad Prism 8.0.1	GraphPad	https://www.graphpad.com; RRID: SCR_002798
FlowJo	TreeStar	https://www.flowjo.com; RRID: SCR_008520
Integrative Genomics Browser (IGV) 2.8.2	Broad Institute	http://www.broadinstitute.org/igv/; RRID: SCR_011793
Scaffold proteome software 4.11.0	Proteome software	http://www.proteomesoftware.com/products/scaffold/; RRID: SCR_014345
Excel	Microsoft	https://products.office.com/
HiC-pro 2.11.1 pipeline	Servant et al., 2015	N/A
Intervene v0.6.4	Khan and Mathelier, 2017	N/A
HiCUP (v0.7.4) pipeline	Wingett et al., 2015	N/A
Bowtie2 v2.3.5.1	Langmead and Salzberg, 2012	N/A
HISAT2 v2.1.0	Kim et al., 2019	N/A
MACS2	Zhang et al., 2008	N/A
NGS plot v2.63	Shen et al., 2014	N/A
Seura” R package	Stuart et al., 2019	N/A

## Highlights

- KSHV episomes tether to host chromosomes at CHD4 enriched regions
- CHD4-LANA complex binds to the PAN RNA promoter and terminal repeat regions
- The viral long non-coding RNA sequesters CHD4 from KSHV genomes during reactivation
- CHD4 facilitates KSHV to establish latency and inhibits reactivation

## References

[R1] Aneja KK, and Yuan Y (2017). Reactivation and lytic replication of kaposi’s sarcoma-associated herpesvirus: an update. Front. Microbiol 8, 613. 10.3389/fmicb.2017.00613. 28473805 PMC5397509

[R2] Arends T, Dege C, Bortnick A, Danhorn T, Knapp JR, Jia H, Harmacek L, Fleenor CJ, Straign D, Walton K, (2019). CHD4 is essential for transcriptional repression and lineage progression in B lymphopoiesis. Proc. Natl. Acad. Sci.U S A 116, 10927–10936. 10.1073/pnas.1821301116. 31085655 PMC6561196

[R3] Arnold PR, Wells AD, and Li XC (2020). Diversity and emerging roles of enhancer RNA in regulation of gene expression and cell fate. Front. Cell Dev. Biol 7, 377. 10.3389/fcell.2019.00377. 31993419 PMC6971116

[R4] Ballestas ME, and Kaye KM (2011). The latency-associated nuclear antigen, a multifunctional protein central to Kaposi’s sarcoma-associated herpesvirus latency. Future Microbiol. 6, 1399–1413. 10.2217/fmb.11.137. 22122438 PMC3857968

[R5] Barbera AJ, Chodaparambil JV, Kelley-Clarke B, Joukov V, Walter JC, Luger K, and Kaye KM (2006). The nucleosomal surface as a docking station for Kaposi’s sarcoma herpesvirus LANA. Science 311, 856–861. 10.1126/science.1120541. 16469929

[R6] Barisic D, Stadler MB, Iurlaro M, and Schubeler D (2019). Mammalian ISWI and SWI/SNF selectively mediate binding of distinct transcription factors. Nature 569, 136–140. 10.1038/s41586-019-1115-5. 30996347 PMC6522387

[R7] Belaghzal H, Dekker J, and Gibcus JH (2017). Hi-C 2.0: an optimized Hi-C procedure for high-resolution genome-wide mapping of chromosome conformation. Methods 123, 56–65. 10.1016/j.ymeth.2017.04.004. 28435001 PMC5522765

[R8] Branon TC, Bosch JA, Sanchez AD, Udeshi ND, Svinkina T, Carr SA, Feldman JL, Perrimon N, and Ting AY (2018). Efficient proximity labeling in living cells and organisms with TurboID. Nat. Biotechnol 36, 880–887. 10.1038/nbt.4201. 30125270 PMC6126969

[R9] Brulois KF, Chang H, Lee ASY, Ensser A, Wong LY, Toth Z, Lee SH, Lee HR, Myoung J, Ganem D, (2012). Construction and manipulation of a new Kaposi’s sarcoma-associated herpesvirus bacterial artificial chromosome clone. J. Virol 86, 9708–9720. 10.1128/jvi.01019-12. 22740391 PMC3446615

[R10] Campbell M, Kim KY, Chang PC, Huerta S, Shevchenko B, Wang DH, Izumiya C, Kung HJ, and Izumiya Y (2014). A lytic viral long noncoding RNA modulates the function of a latent protein. J. Virol 88, 1843–1848. 10.1128/jvi.03251-13. 24257619 PMC3911622

[R11] Campbell M, Watanabe T, Nakano K, Davis RR, Lyu Y, Tepper CG, Durbin-Johnson B, Fujimuro M, and Izumiya Y (2018). KSHV episomes reveal dynamic chromatin loop formation with domain-specific gene regulation. Nat. Commun 9, 49. 10.1038/s41467-017-02089-9. 29302027 PMC5754359

[R12] Carbone A, Gloghini A, Cozzi MR, Capello D, Steffan A, Monini P, De Marco L, and Gaidano G (2000). Expression of MUM1/IRF4 selectively clusters with primary effusion lymphoma among lymphomatous effusions: implications for disease histogenesis and pathogenesis. Br. J. Haematol 111, 247–257. 10.1046/j.1365-2141.2000.02329.x. 11091208

[R13] Cesarman E, and Knowles DM (1999). The role of Kaposi’s sarcoma-associated herpesvirus (KSHV/HHV-8) in lymphoproliferative diseases. Semin. Cancer Biol 9, 165–174. 10.1006/scbi.1998.0118. 10343068

[R14] Cesarman E, Moore PS, Rao PH, Inghirami G, Knowles DM, and Chang Y (1995). In vitro establishment and characterization of two acquired immunodeficiency syndrome-related lymphoma cell lines (BC-1 and BC-2) containing Kaposi’s sarcoma-associated herpesvirus-like (KSHV) DNA sequences. Blood 86, 2708–2714. 10.1182/blood.v86.7.2708.bloodjournal8672708. 7670109

[R15] Chang Y, Cesarman E, Pessin MS, Lee F, Culpepper J, Knowles DM, and Moore PS (1994). Identification of herpesvirus-like DNA sequences in AIDS-sssociated kaposi’s sarcoma. Science 266, 1865–1869. 10.1126/science.7997879. 7997879

[R16] Clarkson CT, Deeks EA, Samarista R, Mamayusupova H, Zhurkin VB, and Teif VB (2019). CTCF-dependent chromatin boundaries formed by asymmetric nucleosome arrays with decreased linker length. Nucleic Acids Res 47, 11181–11196. 10.1093/nar/gkz908. 31665434 PMC6868436

[R17] Coe BP, Stessman HAF, Sulovari A, Geisheker MR, Bakken TE, Lake AM, Dougherty JD, Lein ES, Hormozdiari F, Bernier RA, and Eichler EE (2019). Neurodevelopmental disease genes implicated by de novo mutation and copy number variation morbidity. Nat. Genet 51, 106–116. 10.1038/s41588-018-0288-4. 30559488 PMC6309590

[R18] Correia B, Cerqueira SA, Beauchemin C, Pires de Miranda M, Li S, Ponnusamy R, Rodrigues L, Schneider TR, Carrondo MA, Kaye KM, (2013). Crystal structure of the gamma-2 herpesvirus LANA DNA binding domain identifies charged surface residues which impact viral latency. PLoS Pathog. 9, e1003673. 10.1371/journal.ppat.1003673. 24146618 PMC3798461

[R19] Domsic JF, Chen HS, Lu F, Marmorstein R, and Lieberman PM (2013). Molecular basis for oligomeric-DNA binding and episome maintenance by KSHV LANA. PLoS Pathog. 9, e1003672. 10.1371/journal.ppat.1003672. 24146617 PMC3798644

[R20] Dremel SE, and DeLuca NA (2019). Herpes simplex viral nucleoprotein creates a competitive transcriptional environment facilitating robust viral transcription and host shut off. Elife 8, e51109. 10.7554/elife.51109. 31638576 PMC6805162

[R21] Durand NC, Robinson JT, Shamim MS, Machol I, Mesirov JP, Lander ES, and Aiden EL (2016). Juicebox provides a visualization system for Hi-C contact maps with unlimited zoom. Cell Syst. 3, 99–101. 10.1016/j.cels.2015.07.012. 27467250 PMC5596920

[R22] Ellison TJ, Izumiya Y, Izumiya C, Luciw PA, and Kung HJ (2009). A comprehensive analysis of recruitment and transactivation potential of K-Rta and K-bZIP during reactivation of Kaposi’s sarcoma-associated herpesvirus. Virology 387, 76–88. 10.1016/j.virol.2009.02.016. 19269659 PMC4327937

[R23] Fakhari FD, and Dittmer DP (2002). Charting latency transcripts in Kaposi’s sarcoma-associated herpesvirus by whole-genome real-time quantitative PCR. J. Virol 76, 6213–6223. 10.1128/jvi.76.12.6213-6223.2002. 12021355 PMC136228

[R24] Farnung L, Ochmann M, and Cramer P (2020). Nucleosome-CHD4 chromatin remodeler structure maps human disease mutations. Elife 9, e56178. 10.7554/elife.56178. 32543371 PMC7338049

[R25] Freese NH, Norris DC, and Loraine AE (2016). Integrated genome browser: visual analytics platform for genomics. Bioinformatics 32, 2089–2095. 10.1093/bioinformatics/btw069. 27153568 PMC4937187

[R26] Ganem D (2010). KSHV and the pathogenesis of Kaposi sarcoma: listening to human biology and medicine. J. Clin. Invest 120, 939–949. 10.1172/jci40567. 20364091 PMC2847423

[R27] Garber AC, Shu MA, Hu J, and Renne R (2001). DNA binding and modulation of gene expression by the latency-associated nuclear antigen of Kaposi’s sarcoma-associated herpesvirus. J. Virol 75, 7882–7892. 10.1128/jvi.75.17.7882-7892.2001. 11483733 PMC115032

[R28] Goodman JV, Yamada T, Yang Y, Kong L, Wu DY, Zhao G, Gabel HW, and Bonni A (2020). The chromatin remodeling enzyme Chd4 regulates genome architecture in the mouse brain. Nat. Commun 11, 3419. 10.1038/s41467-020-17065-z. 32647123 PMC7347877

[R29] Gunther T, Frohlich J, Herrde C, Ohno S, Burkhardt L, Adler H, and Grundhoff A (2019). A comparative epigenome analysis of gammaherpesviruses suggests cis-acting sequence features as critical mediators of rapid polycomb recruitment. PLoS Pathog. 15, e1007838. 10.1371/journal.ppat.1007838. 31671162 PMC6932816

[R30] Gunther T, and Grundhoff A (2010). The epigenetic landscape of latent Kaposi sarcoma-associated herpesvirus genomes. PLoS Pathog. 6, e1000935. 10.1371/journal.ppat.1000935. 20532208 PMC2880564

[R31] Hansen AS, Hsieh THS, Cattoglio C, Pustova I, Saldana-Meyer R, Reinberg D, Darzacq X, and Tjian R (2019). Distinct classes of chromatin loops revealed by deletion of an RNA-binding region in CTCF. Mol. Cell 76, 395–411.e13. 10.1016/j.molcel.2019.07.039. 31522987 PMC7251926

[R32] Heinz S, Benner C, Spann N, Bertolino E, Lin YC, Laslo P, Cheng JX, Murre C, Singh H, and Glass CK (2010). Simple combinations of lineage-determining transcription factors prime cis-regulatory elements required for macrophage and B cell identities. Mol. Cell 38, 576–589. 10.1016/j.molcel.2010.05.004. 20513432 PMC2898526

[R33] Hellert J, Weidner-Glunde M, Krausze J, Richter U, Adler H, Fedorov R, Pietrek M, Ruckert J, Ritter C, Schulz TF, and Luhrs T (2013). A structural basis for BRD2/4-mediated host chromatin interaction and oligomer assembly of Kaposi sarcoma-associated herpesvirus and murine gammaherpesvirus LANA proteins. PLoS Pathog. 9, e1003640. 10.1371/journal.ppat.1003640. 24146614 PMC3798688

[R34] Hu J, Garber AC, and Renne R (2002). The latency-associated nuclear antigen of Kaposi’s sarcoma-associated herpesvirus supports latent DNA replication in dividing cells. J. Virol 76, 11677–11687. 10.1128/jvi.76.22.11677-11687.2002. 12388727 PMC136756

[R35] Isoda T, Moore AJ, He Z, Chandra V, Aida M, Denholtz M, Piet van Hamburg J, Fisch KM, Chang AN, Fahl SP, (2017). Non-coding transcription instructs chromatin folding and compartmentalization to dictate enhancer-promoter communication and T cell fate. Cell 171, 103–119.e18. 10.1016/j.cell.2017.09.001. 28938112 PMC5621651

[R36] Izumiya Y, Ellison TJ, Yeh ETH, Jung JU, Luciw PA, and Kung HJ (2005). Kaposi’s sarcoma-associated herpesvirus K-bZIP represses gene transcription via SUMO modification. J. Virol 79, 9912–9925. 10.1128/jvi.79.15.9912-9925.2005. 16014952 PMC1181544

[R37] Izumiya Y, Izumiya C, Hsia D, Ellison TJ, Luciw PA, and Kung HJ (2009). NF-κB serves as a cellular sensor of kaposi’s sarcoma-associated herpesvirus latency and negatively regulates K-rta by antagonizing the RBP-Jκ coactivator. J. Virol 83, 4435–4446. 10.1128/jvi.01999-08. 19244329 PMC2668470

[R38] Jiang S, Zhou H, Liang J, Gerdt C, Wang C, Ke L, Schmidt SCS, Narita Y, Ma Y, Wang S, (2017). The epstein-barr virus regulome in lymphoblastoid cells. Cell Host Microbe 22, 561–573.e4. 10.1016/j.chom.2017.09.001. 29024646 PMC5662195

[R39] Jung I, Schmitt A, Diao Y, Lee AJ, Liu T, Yang D, Tan C, Eom J, Chan M, Chee S, (2019). A compendium of promoter-centered longrange chromatin interactions in the human genome. Nat. Genet 51, 1442–1449. 10.1038/s41588-019-0494-8. 31501517 PMC6778519

[R40] Kaaij LJT, Mohn F, van der Weide RH, de Wit E, and Buhler M (2019). The ChAHP complex counteracts chromatin looping at CTCF sites that emerged from SINE expansions in mouse. Cell 178, 1437–1451.e14. 10.1016/j.cell.2019.08.007. 31491387

[R41] Kedes DH, Lagunoff M, Renne R, and Ganem D (1997). Identification of the gene encoding the major latency-associated nuclear antigen of the Kaposi’s sarcoma-associated herpesvirus. J. Clin. Invest 100, 2606–2610. 10.1172/jci119804. 9366576 PMC508462

[R42] Khan A, and Mathelier A (2017). Intervene: a tool for intersection and visualization of multiple gene or genomic region sets. BMC Bioinf. 18, 287. 10.1186/s12859-017-1708-7. PMC5452382 28569135

[R43] Kim D, Paggi JM, Park C, Bennett C, and Salzberg SL (2019). Graph-based genome alignment and genotyping with HISAT2 and HISAT-genotype. Nat. Biotechnol 37, 907–915. 10.1038/s41587-019-0201-4. 31375807 PMC7605509

[R44] Kim KY, Huerta SB, Izumiya C, Wang DH, Martinez A, Shevchenko B, Kung HJ, Campbell M, and Izumiya Y (2013). Kaposi’s sarcoma-associated herpesvirus (KSHV) latency-associated nuclear antigen regulates the KSHV epigenome by association with the histone demethylase KDM3A. J. Virol 87, 6782–6793. 10.1128/jvi.00011-13. 23576503 PMC3676133

[R45] Kovac K, Sauer A, Macinkovic I, Awe S, Finkernagel F, Hoffmeister H, Fuchs A, Muller R, Rathke C, Langst G, and Brehm A (2018). Tumour-associated missense mutations in the dMi-2 ATPase alters nucleosome remodelling properties in a mutation-specific manner. Nat. Commun 9, 2112. 10.1038/s41467-018-04503-2. 29844320 PMC5974244

[R46] Kumar A, Salemi M, Bhullar R, Guevara-Plunkett S, Lyu Y, Wang KH, Izumiya C, Campbell M, Nakajima KI, and Izumiya Y (2021). Proximity biotin labeling reveals kaposi’s sarcoma-associated herpesvirus interferon regulatory factor networks. J. Virol 95, e02049–e02120. 10.1128/jvi.02049-20. 33597212 PMC8104114

[R47] Lagunoff M, and Ganem D (1997). The structure and coding organization of the genomic termini of kaposi’s sarcoma-associated herpesvirus (human herpesvirus 8). Virology 236, 147–154. 10.1006/viro.1997.8713. 9299627

[R48] Langmead B, and Salzberg SL (2012). Fast gapped-read alignment with Bowtie 2. Nat. Methods 9, 357–359. 10.1038/nmeth.1923. 22388286 PMC3322381

[R49] Le Gallo M, O’Hara AJ, Rudd ML, Urick ME, Hansen NF, O’Neil NJ, Price JC, Zhang S, England BM, Godwin AK, (2012). Exome sequencing of serous endometrial tumors identifies recurrent somatic mutations in chromatin-remodeling and ubiquitin ligase complex genes. Nat. Genet 44, 1310–1315. 10.1038/ng.2455. 23104009 PMC3515204

[R50] Lim C, Lee D, Seo T, Choi C, and Choe J (2003). Latency-associated nuclear antigen of Kaposi’s sarcoma-associated herpesvirus functionally interacts with heterochromatin protein 1. J. Biol. Chem 278, 7397–7405. 10.1074/jbc.m211912200. 12486118

[R51] Lu P, Li M, Zhang D, and Jiang W (2021). Lnc-ing pluripotency maintenance and early differentiation in human pluripotent stem cells. FASEB J. 35, e21438. 10.1096/fj.202002278r. 33749897

[R52] Ma Y, Walsh MJ, Bernhardt K, Ashbaugh CW, Trudeau SJ, Ashbaugh IY, Jiang S, Jiang C, Zhao B, Root DE, (2017). CRISPR/Cas9 screens reveal epstein-barr virus-transformed B cell host dependency factors. Cell Host Microbe 21, 580–591.e7. 10.1016/j.chom.2017.04.005. 28494239 PMC8938989

[R53] Majerciak V, Pripuzova N, McCoy JP, Gao SJ, and Zheng ZM (2007). Targeted disruption of kaposi’s sarcoma-associated herpesvirus ORF57 in the viral genome is detrimental for the expression of ORF59, K8a, and K8.1 and the production of infectious virus. J. Virol 81, 1062–1071. 10.1128/jvi.01558-06. 17108026 PMC1797518

[R54] Manzano M, Gunther T, Ju H, Nicholas J, Bartom ET, Grundhoff A, and Gottwein E (2020). Kaposi’s sarcoma-associated herpesvirus drives a superenhancer-mediated survival gene expression program in primary effusion lymphoma. mBio 11, e01457–e01520. 10.1128/mbio.01457-20. 32843547 PMC7448273

[R55] Massimelli MJ, Kang JG, Majerciak V, Le SY, Liewehr DJ, Steinberg SM, and Zheng ZM (2011). Stability of a long noncoding viral RNA depends on a 9-nt core element at the RNA 5’ end to interact with viral ORF57 and cellular PABPC1. Int. J. Biol. Sci 7, 1145–1160. 10.7150/ijbs.7.1145. PMC3204405 22043172

[R56] Matsumura S, Persson LM, Wong L, and Wilson AC (2010). The latency-associated nuclear antigen interacts with MeCP2 and nucleosomes through separate domains. J. Virol 84, 2318–2330. 10.1128/jvi.01097-09. 20032179 PMC2820923

[R57] McSwiggen DT, Hansen AS, Teves SS, Marie-Nelly H, Hao Y, Heckert AB, Umemoto KK, Dugast-Darzacq C, Tjian R, and Darzacq X (2019). Evidence for DNA-mediated nuclear compartmentalization distinct from phase separation. Elife 8, e47098. 10.7554/elife.47098. 31038454 PMC6522219

[R58] Mesri EA, Cesarman E, and Boshoff C (2010). Kaposi’s sarcoma and its associated herpesvirus. Nat. Rev. Cancer 10, 707–719. 10.1038/nrc2888. 20865011 PMC4721662

[R59] Morra R, Lee BM, Shaw H, Tuma R, and Mancini EJ (2012). Concerted action of the PHD, chromo and motor domains regulates the human chromatin remodelling ATPase CHD4. FEBS Lett. 586, 2513–2521. 10.1016/j.febslet.2012.06.017. 22749909 PMC3476528

[R60] Myoung J, and Ganem D (2011). Generation of a doxycycline-inducible KSHV producer cell line of endothelial origin: maintenance of tight latency with efficient reactivation upon induction. J Virol Methods. 174(1–2): 12–21. 10.1016/j.jviromet.2011.03.012. 21419799 PMC3095772

[R61] Naik NG, Nguyen TH, Roberts L, Fischer LT, Glickman K, Golas G, Papp B, and Toth Z (2020). Epigenetic factor siRNA screen during primary KSHV infection identifies novel host restriction factors for the lytic cycle of KSHV. PLoS Pathog. 16, e1008268. 10.1371/journal.ppat.1008268. 31923286 PMC6977772

[R62] Nair SJ, Yang L, Meluzzi D, Oh S, Yang F, Friedman MJ, Wang S, Suter T, Alshareedah I, Gamliel A, (2019). Phase separation of ligand-activated enhancers licenses cooperative chromosomal enhancer assembly. Nat. Struct. Mol. Biol 26, 193–203. 10.1038/s41594019-0190-5. 30833784 PMC6709854

[R63] Nakamura H, Lu M, Gwack Y, Souvlis J, Zeichner SL, and Jung JU (2003). Global Changes in Kaposi’s Sarcoma-Associated Virus Gene Expression Patterns following Expression of a Tetracycline-Inducible Rta Transactivator. J. Virol 77(7): 4205–4220. 10.1128/JVI.77.7.4205-4220.2003. 12634378 PMC150665

[R64] Nesvizhskii AI, Keller A, Kolker E, and Aebersold R (2003). A statistical model for identifying proteins by tandem mass spectrometry. Anal. Chem 75, 4646–4658. 10.1021/ac0341261. 14632076

[R65] Ostapcuk V, Mohn F, Carl SH, Basters A, Hess D, Iesmantavicius V, Lampersberger L, Flemr M, Pandey A, Thoma NH, (2018). Activity-dependent neuroprotective protein recruits HP1 and CHD4 to control lineage-specifying genes. Nature 557, 739–743. 10.1038/s41586-018-0153-8. 29795351

[R66] Ottinger M, Christalla T, Nathan K, Brinkmann MM, Viejo-Borbolla A, and Schulz TF (2006). Kaposi’s sarcoma-associated herpesvirus LANA-1 interacts with the short variant of BRD4 and releases cells from a BRD4- and BRD2/RING3-induced G1 cell cycle arrest. J. Virol 80, 10772–10786. 10.1128/jvi.00804-06. 16928766 PMC1641788

[R67] Piolot T, Tramier M, Coppey M, Nicolas JC, and Marechal V (2001). Close but distinct regions of human herpesvirus 8 latency-associated nuclear antigen 1 are responsible for nuclear targeting and binding to human mitotic chromosomes. J. Virol 75, 3948–3959. 10.1128/jvi.75.8.3948-3959.2001. 11264383 PMC114885

[R68] Purushothaman P, Thakker S, and Verma SC (2015). Transcriptome analysis of Kaposi’s sarcoma-associated herpesvirus during de novo primary infection of human B and endothelial cells. J. Virol 89, 3093–3111. 10.1128/jvi.02507-14. 25552714 PMC4337554

[R69] Rao SS, Huntley MH, Durand NC, Stamenova EK, Bochkov ID, Robinson JT, Sanborn AL, Machol I, Omer AD, Lander ES, and Aiden E (2014). A 3D map of the human genome at kilobase resolution reveals principles of chromatin looping. Cell 159, 1665–1680. 10.1016/j.cell.2014.11.021. 25497547 PMC5635824

[R70] Renne R, Blackbourn D, Whitby D, Levy J, and Ganem D (1998). Limited Transmission of Kaposi’s Sarcoma-Associated Herpesvirus in Cultured Cells. J. Virol 72(6): 5182–5188. 10.1128/jvi.72.6.5182-5188.1998. 9573290 PMC110093

[R71] Rossetto CC, and Pari G (2012). KSHV PAN RNA associates with demethylases UTX and JMJD3 to activate lytic replication through a physical interaction with the virus genome. PLoS Pathog. 8, e1002680. 10.1371/journal.ppat.1002680. 22589717 PMC3349751

[R72] Rossetto CC, Tarrant-Elorza M, Verma S, Purushothaman P, and Pari GS (2013). Regulation of viral and cellular gene expression by Kaposi’s sarcoma-associated herpesvirus polyadenylated nuclear RNA. J. Virol 87, 5540–5553. 10.1128/jvi.03111-12. 23468496 PMC3648157

[R73] Saldana-Meyer R, Rodriguez-Hernaez J, Escobar T, Nishana M, Jacome-Lopez K, Nora EP, Bruneau BG, Tsirigos A, Furlan-Magaril M, Skok J, and Reinberg D (2019). RNA interactions are essential for CTCF-mediated genome organization. Mol. Cell 76, 412–422.e5. 10.1016/j.molcel.2019.08.015. 31522988 PMC7195841

[R74] Schmitt AD, Hu M, and Ren B (2016). Genome-wide mapping and analysis of chromosome architecture. Nat. Rev. Mol. Cell Biol 17, 743–755. 10.1038/nrm.2016.104. 27580841 PMC5763923

[R75] Schoenfelder S, Javierre B-M, Furlan-Magaril M, Wingett SW, and Fraser P (2018). Promoter capture Hi-C: high-resolution, genome-wide profiling of promoter interactions. JoVE 136, 57320. 10.3791/57320. PMC6102006 30010637

[R76] Schulz TF (2006). The pleiotropic effects of Kaposi’s sarcoma herpesvirus. J. Pathol 208, 187–198. 10.1002/path.1904. 16362980

[R77] Servant N, Varoquaux N, Lajoie BR, Viara E, Chen CJ, Vert JP, Heard E, Dekker J, and Barillot E (2015). HiC-Pro: an optimized and flexible pipeline for Hi-C data processing. Genome Biol 16, 259. 10.1186/s13059-015-0831-x. 26619908 PMC4665391

[R78] Shen L, Shao N, Liu X, and Nestler E (2014). ngs.plot: Quick mining and visualization of next-generation sequencing data by integrating genomic databases. BMC Genomics 15, 284. 10.1186/1471-2164-15-284. 24735413 PMC4028082

[R79] Si H, Verma SC, Lampson MA, Cai Q, and Robertson ES (2008). Kaposi’s sarcoma-associated herpesvirus-encoded LANA can interact with the nuclear mitotic apparatus protein to regulate genome maintenance and segregation. J. Virol 82, 6734–6746. 10.1128/jvi.00342-08. 18417561 PMC2447046

[R80] Sifrim A, Hitz MP, Wilsdon A, Breckpot J, Turki SH, Thienpont B, McRae J, Fitzgerald TW, Singh T, Swaminathan GJ, ; the UK10K Consortium; the Deciphering Developmental Disorders Study (2016). Distinct genetic architectures for syndromic and nonsyndromic congenital heart defects identified by exome sequencing. Nat. Genet 48, 1060–1065. 10.1038/ng.3627. 27479907 PMC5988037

[R81] Skene PJ, and Henikoff S (2017). An efficient targeted nuclease strategy for high-resolution mapping of DNA binding sites. Elife 6, e21856. 10.7554/elife.21856. 28079019 PMC5310842

[R82] Song MJ, Brown HJ, Wu TT, and Sun R (2001). Transcription activation of polyadenylated nuclear rna by rta in human herpesvirus 8/Kaposi’s sarcoma-associated herpesvirus. J. Virol 75, 3129–3140. 10.1128/jvi.75.7.3129-3140.2001. 11238840 PMC114107

[R83] Stedman W, Deng Z, Lu F, and Lieberman PM (2004). ORC, MCM, and histone hyperacetylation at the Kaposi’s sarcoma-associated herpesvirus latent replication origin. J. Virol 78, 12566–12575. 10.1128/jvi.78.22.12566-12575.2004. 15507644 PMC525046

[R84] Strahan RC, McDowell-Sargent M, Uppal T, Purushothaman P, and Verma SC (2017). KSHV encoded ORF59 modulates histone arginine methylation of the viral genome to promote viral reactivation. PLoS Pathog. 13, e1006482. 10.1371/journal.ppat.1006482. 28678843 PMC5513536

[R85] Stuart T, Butler A, Hoffman P, Hafemeister C, Papalexi E, Mauck WM 3rd, Hao Y, Stoeckius M, Smibert P, and Satija R (2019). Comprehensive integration of single-cell data. Cell 177, 1888–1902.e21. 10.1016/j.cell.2019.05.031. 31178118 PMC6687398

[R86] Sun R, Lin SF, Gradoville L, and Miller G (1996). Polyadenylylated nuclear RNA encoded by Kaposi sarcoma-associated herpesvirus. Proc. Natl. Acad. Sci. U S A 93, 11883–11888. 10.1073/pnas.93.21.11883. 8876232 PMC38153

[R87] Sun X, Yu W, Li L, and Sun Y (2020). ADNP controls gene expression through local chromatin architecture by association with BRG1 and CHD4. Front. Cell Dev. Biol 8, 553. 10.3389/fcell.2020.00553. 32714933 PMC7341970

[R88] Tischer BK, Smith GA, and Osterrieder N (2010). En passant mutagenesis: a two step markerless red recombination system. Methods Mol. Biol 634, 421–430. 10.1007/978-1-60761-652-8_30. 20677001

[R89] Toth Z, Maglinte DT, Lee SH, Lee HR, Wong LY, Brulois KF, Lee S, Buckley JD, Laird PW, Marquez VE, and Jung JU (2010). Epigenetic analysis of KSHV latent and lytic genomes. PLoS Pathog. 6, e1001013. 10.1371/journal.ppat.1001013. 20661424 PMC2908616

[R90] Verma SC, Cai Q, Kreider E, Lu J, and Robertson ES (2013). Comprehensive analysis of LANA interacting proteins essential for viral genome tethering and persistence. PLoS One 8, e74662. 10.1371/journal.pone.0074662. 24040311 PMC3770571

[R91] Verma SC, Choudhuri T, Kaul R, and Robertson ES (2006). Latency-associated nuclear antigen (LANA) of Kaposi’s sarcoma-associated herpesvirus interacts with origin recognition complexes at the LANA binding sequence within the terminal repeats. J. Virol 80, 2243–2256. 10.1128/jvi.80.5.2243-2256.2006. 16474132 PMC1395374

[R92] Wang C, Zhang L, Ke L, Ding W, Jiang S, Li D, Narita Y, Hou I, Liang J, Li S, (2020). Primary effusion lymphoma enhancer connectome links super-enhancers to dependency factors. Nat. Commun 11, 6318. 10.1038/s41467-020-20136-w. 33298918 PMC7726151

[R93] Wang N, Wu R, Tang D, and Kang R (2021). The BET family in immunity and disease. Signal. Transduct Target. Ther 6, 23. 10.1038/s41392-020-00384-4. 33462181 PMC7813845

[R94] Weiss K, Lazar HP, Kurolap A, Martinez AF, Paperna T, Cohen L, Smeland MF, Whalen S, Heide S, Keren B, (2020). The CHD4-related syndrome: a comprehensive investigation of the clinical spectrum, genotype-phenotype correlations, and molecular basis. Genet. Med 22, 389–397. 10.1038/s41436-019-0612-0. 31388190 PMC8900827

[R95] Wingett SW, Ewels P, Furlan-Magaril M, Nagano T, Schoenfelder S, Fraser P, and Andrews S (2015). HiCUP: pipeline for mapping and processing Hi-C data. F1000Res. 4, 1310. 10.12688/f1000research.7334.1. 26835000 PMC4706059

[R96] Withers JB, Li ES, Vallery TK, Yario TA, and Steitz JA (2018). Two herpesviral noncoding PAN RNAs are functionally homologous but do not associate with common chromatin loci. PLoS Pathog. 14, e1007389. 10.1371/journal.ppat.1007389. 30383841 PMC6233925

[R97] Wong EL, and Damania B (2005). Linking KSHV to human cancer. Curr. Oncol. Rep 7, 349–356. 10.1007/s11912-005-0061-6. 16091195

[R98] Xiao B, Verma SC, Cai Q, Kaul R, Lu J, Saha A, and Robertson ES (2010). Bub1 and CENP-F can contribute to Kaposi’s sarcoma-associated herpesvirus genome persistence by targeting LANA to kinetochores. J. Virol 84, 9718–9732. 10.1128/jvi.00713-10. 20660191 PMC2937805

[R99] Yan L, Majerciak V, Zheng ZM, and Lan K (2019). Towards better understanding of KSHV life cycle: from transcription and posttranscriptional regulations to pathogenesis. Virol. Sin 34, 135–161. 10.1007/s12250019-00114-3. 31025296 PMC6513836

[R100] You J, Srinivasan V, Denis GV, Harrington WJ Jr., Ballestas ME, Kaye KM, and Howley PM (2006). Kaposi’s sarcoma-associated herpesvirus latency-associated nuclear antigen interacts with bromodomain protein Brd4 on host mitotic chromosomes. J. Virol 80, 8909–8919. 10.1128/jvi.00502-06. 16940503 PMC1563901

[R101] Zhang Y, Liu T, Meyer CA, Eeckhoute J, Johnson DS, Bernstein BE, Nusbaum C, Myers RM, Brown M, Li W, and Liu XS (2008). Model-based analysis of ChIP-seq (MACS). Genome Biol 9, R137. 10.1186/gb-2008-9-9-r137. 18798982 PMC2592715

[R102] Zhao H, Han Z, Liu X, Gu J, Tang F, Wei G, and Jin Y (2017). The chromatin remodeler Chd4 maintains embryonic stem cell identity by controlling pluripotency- and differentiation-associated genes. J. Biol. Chem 292, 8507–8519. 10.1074/jbc.m116.770248. 28298436 PMC5437254

[R103] Zhao S, Choi M, Overton JD, Bellone S, Roque DM, Cocco E, Guzzo F, English DP, Varughese J, Gasparrini S, (2013). Landscape of somatic single-nucleotide and copy-number mutations in uterine serous carcinoma. Proc. Natl. Acad. Sci. U S A 110, 2916–2921. 10.1073/pnas.1222577110. 23359684 PMC3581983

[R104] Zhou Y, Zhou B, Pache L, Chang M, Khodabakhshi AH, Tanaseichuk O, Benner C, and Chanda SK (2019). Metascape provides a biologist-oriented resource for the analysis of systems-level datasets. Nat. Commun 10, 1523. 10.1038/s41467-019-09234-6. 30944313 PMC6447622

