# Supplementary material for: KSHV episome tethering sites on host chromosomes and regulation of latency-lytic switch by CHD4
Source: Cell Rep. Author manuscript; Available in PMC 2022 May 31. (PMC9153692; doi:10.1016/j.celrep.2022.110788)
Supplement: 1 [file NIHMS1806830-supplement-1.pdf]

Cell Reports, Volume *39*

Supplemental information

KSHV episome tethering sites on host chromosomes

and regulation of latency-lytic switch by CHD4

Ashish Kumar, Yuanzhi Lyu, Yuichi Yanagihashi, Chanikarn Chantarasrivong, Vladimir Majerciak, Michelle Salemi, Kang-Hsin Wang, Tomoki Inagaki, Frank Chuang, Ryan R. Davis, Clifford G. Tepper, Kazushi Nakano, Chie Izumiya, Michiko Shimoda, Ken-ichi Nakajima, Alexander Merleev, Zhi-Ming Zheng, Mel Campbell, and Yoshihiro Izumiya

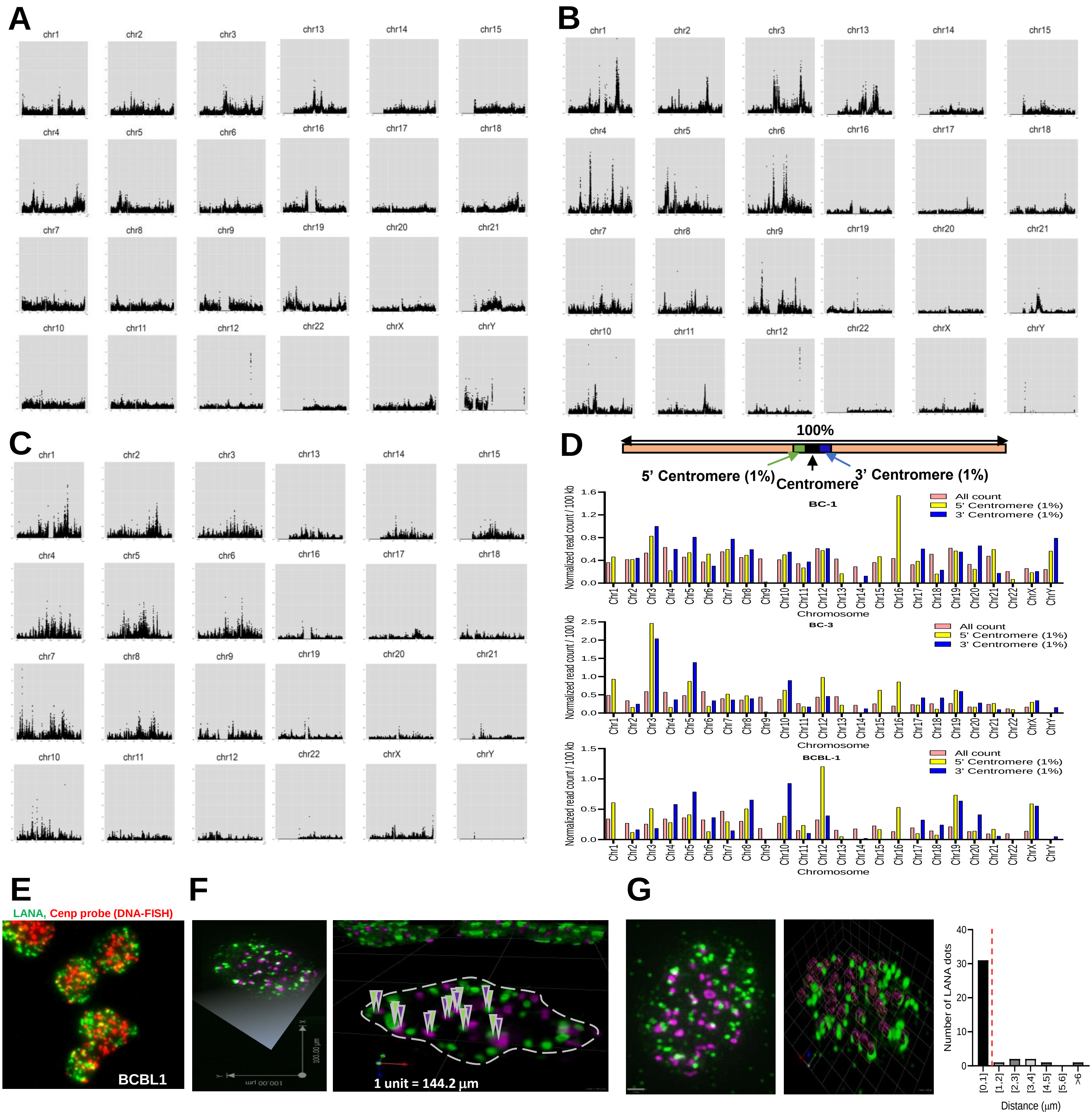

Figure S1. Related to Figure 1. (A-C) CHi-C dot plot. Chi-C reads were mapped on human genome sequence and plotted as dot plots for **(A)** BC-1, **(B)** BC-3 and **(C)** BCBL-1. X: position of human chromosomes, Y: KSHV sequence reads. n=1 biological replicate. **(D)** KSHV episome contacts were calculated in BCBL-1 (top), BC-1 (middle) and BC-3 (bottom) cells for sequence reads in 1% (length of the respective chromosome) of the 5'-and 3'-centromere and compared with the average of the total chromosome read count. **(E)** DNA-FISH was used to visualize the location of the centromere (red) and KSHV episomes were indirectly visualized by staining LANA dots (Green) in BCBL-1 cells. The centromere and LANA were probed using a Cenp probe and anti-LANA antibody respectively with IFA. The two signals were frequently co-localized, and 3D view, and their image analyses are presented in Figure S1F. **(F) Left**: Flat 2D projection of BCBL-1 nucleus with labeled LANA (green) and centromere oligo probes for DNA-FISH (purple). ***Right***: oblique view of same nucleus, displayed as a 3D image stack reveals proximity localization of several LANA signals with centromere. **(G) Mathematic calculation for distance between centromere and LANA dots. Related to Figure 1.** For each cell nucleus, the shortest distance from each centromere (purple) to the nearest LANA dot (green) neighbor was measured from reconstructed 3D image stacks using Volocity Quantitation and compiled as individual histograms. In every case, most distances measured were between 0 and 1 µm – considerably less than the calculated mean (marked by red dashed line). This left-skewed distribution strongly suggests that LANA dots are indeed tethered near centromeric points in the host cell nucleus.

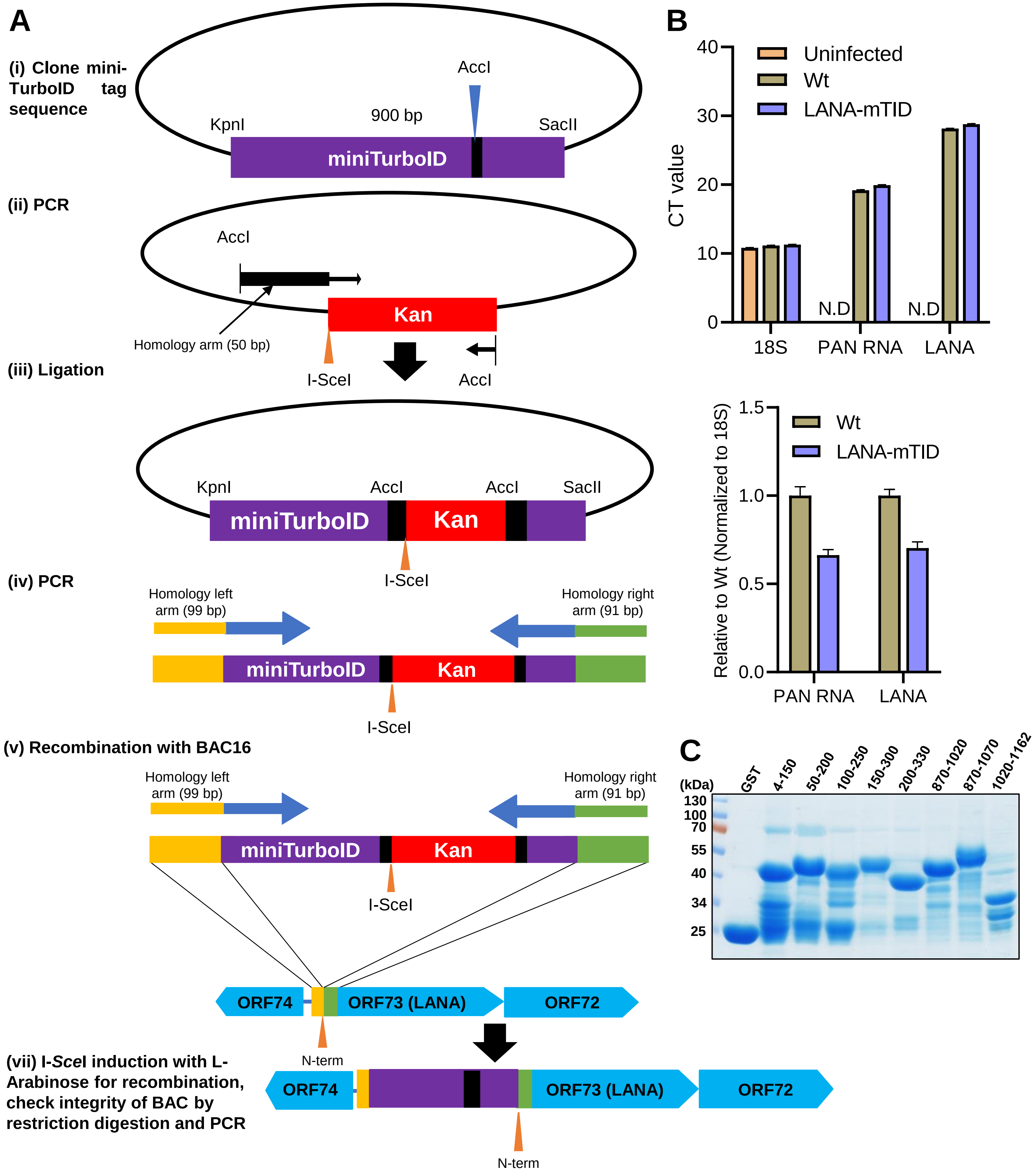

Figure S2. Related to Figure 2. (A) Engineering of mini-TurboID KSHVs. (i-vii) Schematic diagram for construction of KSHV LANA-mTID. Detailed protocol for preparation is written in the Methods section. **(B) Comparison between wt and LANA-mTID KSHV.** 293FT cells were infected with equal amount of wild type (wt) KSHV and LANA-mTID KSHV for 24h. PAN RNA and ORF73 expression was measured. Ct value (Top graph) and relative expression (Bottom graph) are shown. ND: not detected. **(C) Coommasie blue staining**. GST-tagged LANA deletion were prepared from recombinant baculovirus infected Sf9 cells. Coommasie blue staining is shown. Purified GST-tagged LANA deletions were used in **Figure 2E. (B)** n=3 biological replicates, and representative qPCR with technical triplicates is shown. Data are presented as mean ± SD.

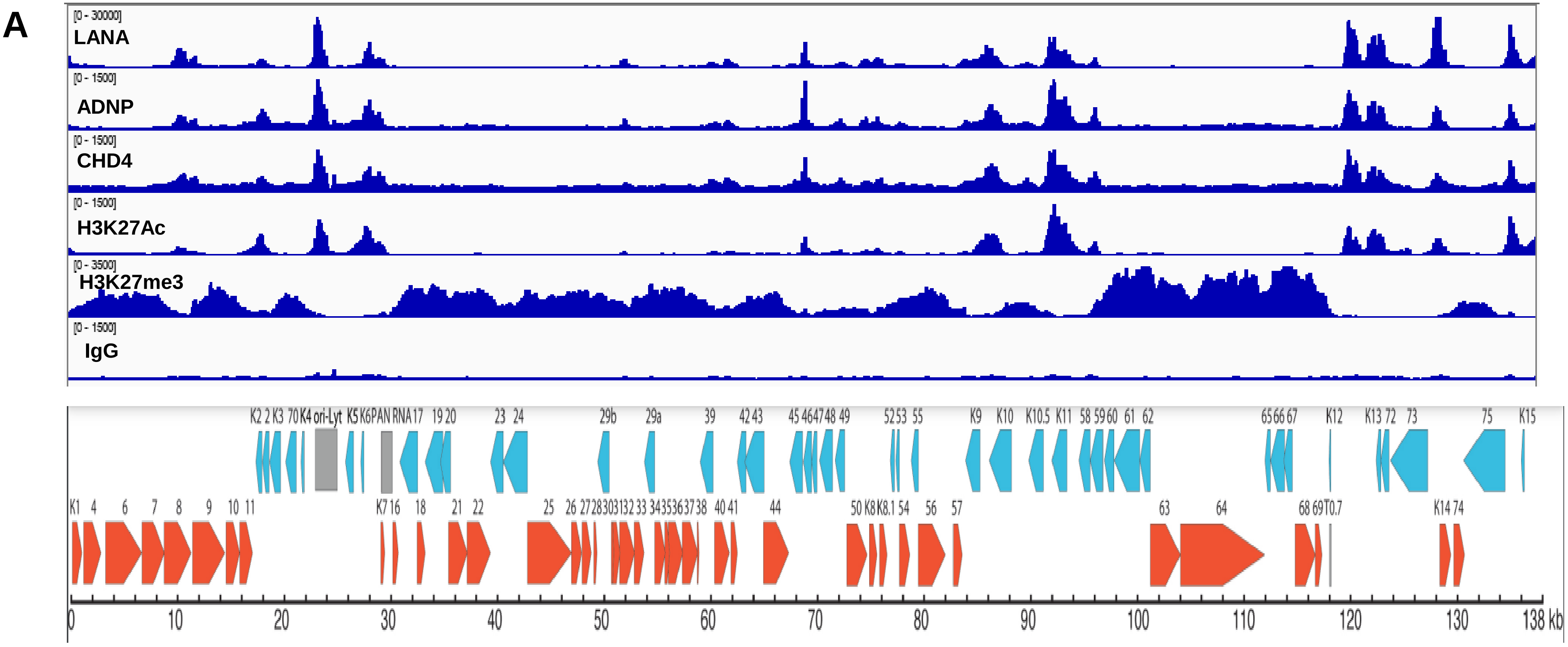

Figure S3. (A) CUT & RUN analysis. Related to Figure 4. The indicated antibodies were used for CUT&RUN in BCBL-1 cells and sequence reads were mapped to the KSHV genome. An IGV snapshot and KSHV genome map are shown (**upper panels**). KSHV open reading frames are also shown (**bottom panel**). n=2 biological replicates for each CUT&RUN, one representative is shown.

A

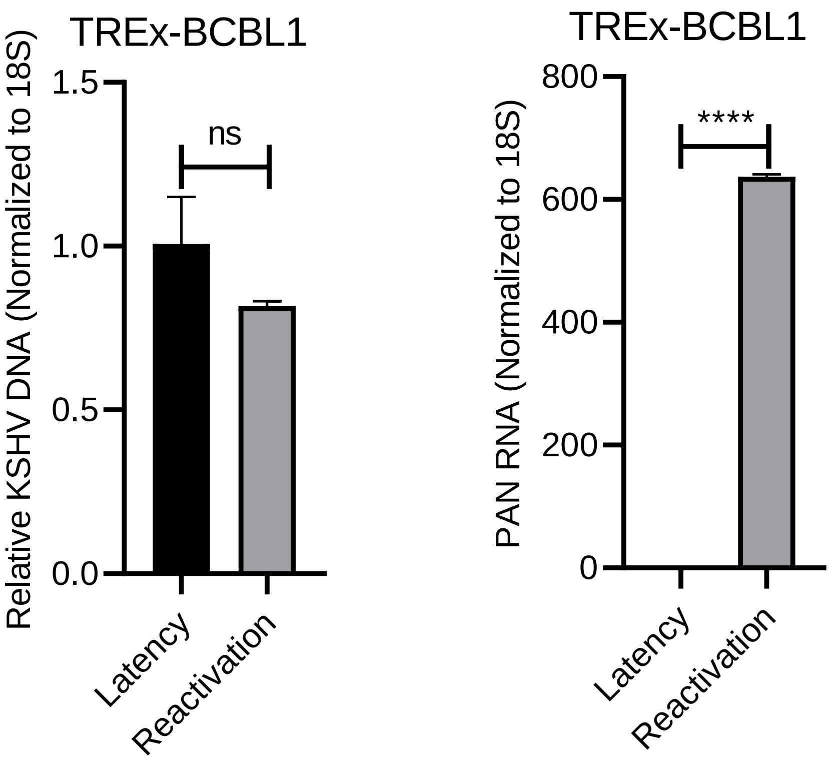

Figure S4. (A) KSHV genomic copy. Related to Figure 4. TREx-BCBL-1 cells were either left unstimulated (latency) or stimulated with doxycycline (1 µg/ml) and TPA (20 nM) (reactivation) for 24 h. The genomic DNA and RNA was isolated, and the viral genomic copy (**left side panel**) and viral gene expression was measured (**right side panel**). n=3 biological replicates, and representative qPCR with technical triplicates is shown. Data are presented as mean ± SD. Unpaired two-tailed student t-test was used to calculate the significance. ****$p<0.0001$, ns: non-significant.

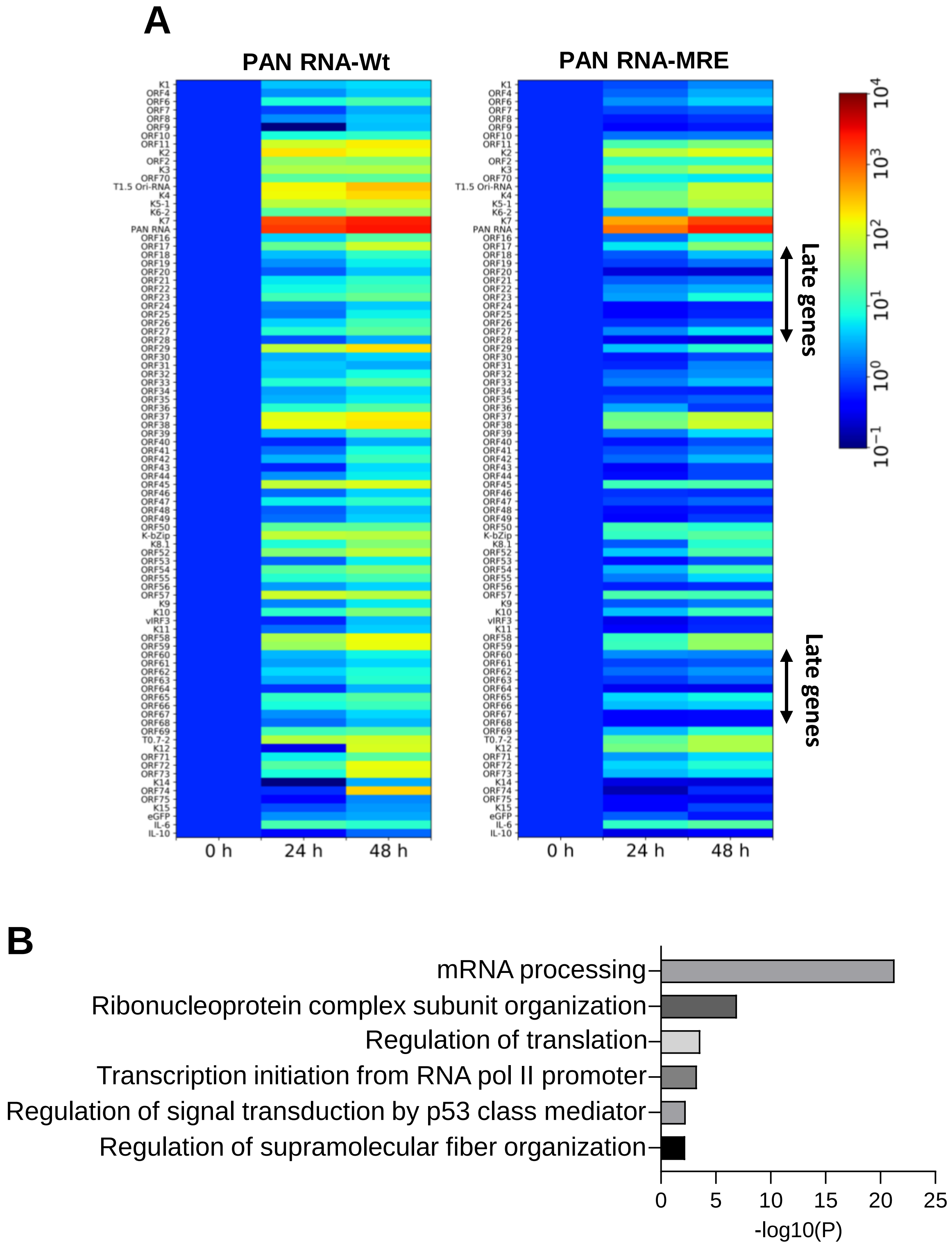

Figure S5. Related to Figure 5. (A) KSHV RT-qPCR arrays. The genome-wide expression of KSHV genes and lncRNAs was examined by PCR arrays and fold activation over 0 hr time point was plotted as heat maps. **(B) Gene Ontology analyses**. Gene Ontology (GO) analysis of 129 PAN RNA-mediated ORF57-interacting proteins was performed using Metascape software. Non-redundant top 6 biological pathways are presented. **(A)** n=2 biological replicates.

A

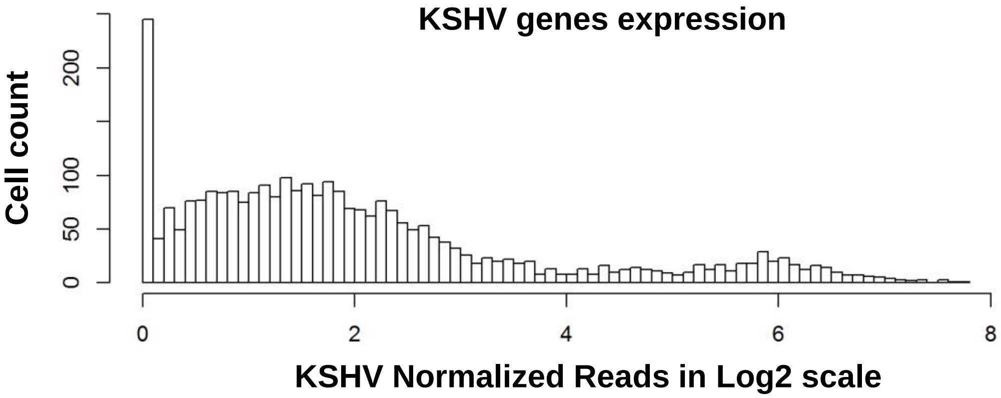

B

Interval	Cells
(-0.001,0.001]	245
(0.001,0.5]	236
(0.5,1]	406
(1,1.5]	439
(1.5,2]	421
(2,2.5]	329
(2.5,3]	214
(3,3.5]	109
(3.5,4]	67
(4,4.5]	55
(4.5,5]	58
(5,5.5]	63
(5.5,6]	96
(6,7.7]	134

C

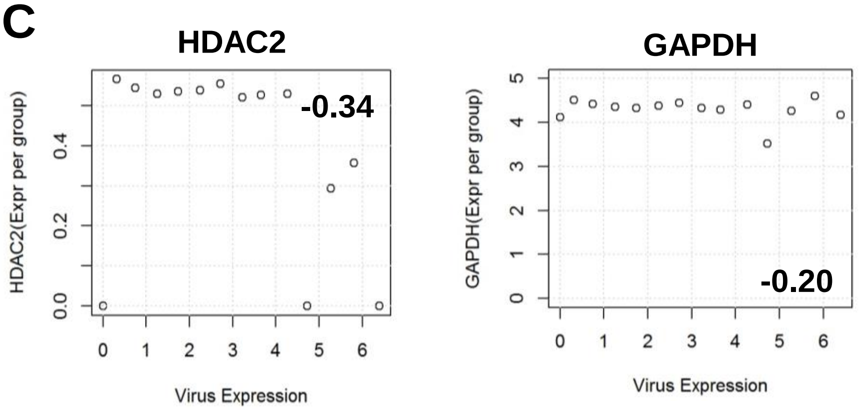

D E

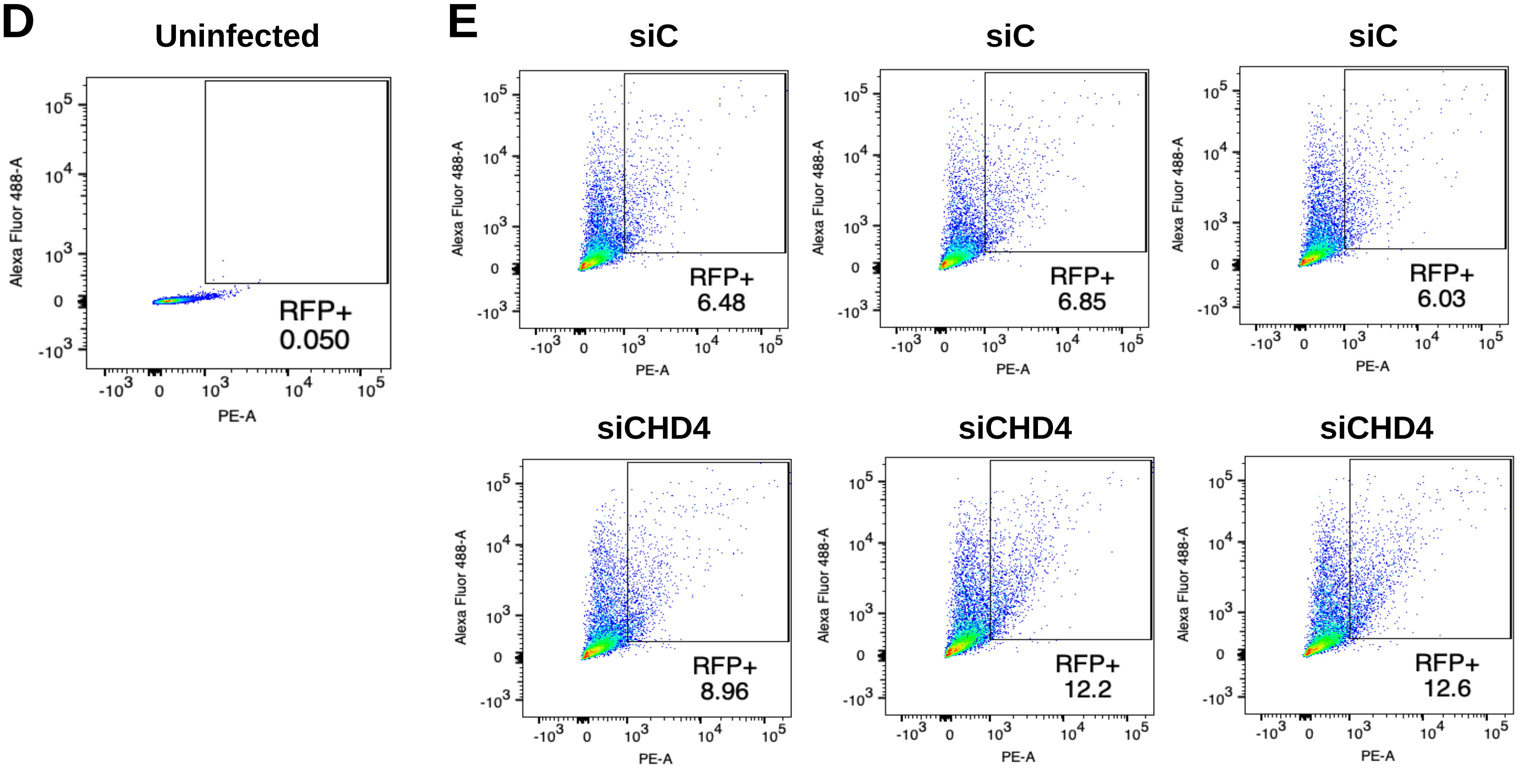

Figure S6. . Related to Figure 6. (A-C) Single cell sequencing. iSLK cells infected with KSHV were reactivated by induction of K-Rta expression from doxycycline inducible promoter for 24 hours. Single cell suspension was prepared, and library were made with 10x genomics platform. **(A)** Histogram showing the distribution of cells per KSHV gene expression. n=1 biological replicate. **(B)** Table showing distribution of cells per KSHV gene expression. Each interval has a good number of cells to estimate gene expression in each interval. Groups showed in the table were used to estimate other gene expression for the downstream correlation analysis. **(C)** Correlation of component of NuRD complex (HDAC2) and GAPDH with KSHV transcripts. **(D-E) Flow Cytometry. (D)** Uninfected cells were used to gate out autofluorescence. **(E)** 293FT cells were transfected with control siRNA (siC) or CHD4 siRNA (siCHD4) for 48 hrs followed by infection with rKSHV.219 virus for 96 hrs. The gating from (D) was applied to siC and siCHD4 samples to calculated the percentage of RFP+ cells. **(E)** n=3 biological replicates.
